# Melatonin and Brassinolide Enhance Cold Tolerance in *Osmanthus fragrans*: Insights from Integrated Physiological and Multi-Omics Analyses

**DOI:** 10.3390/plants15152404

**Published:** 2026-08-06

**Authors:** Hui Xia, Wenxuan Huang, Jingjing Zou, Hongguo Chen, Xuan Cai, Jie Yang, Zeqing Li, Xiangling Zeng, Yuanhang Wu, Yingting Zhang

**Affiliations:** 1 Key Laboratory of National Forestry and Grassland Administration on *Osmanthus fragrans*, Hubei University of Science and Technology, Xianning 437100, China; xiahui625649@163.com (H.X.); 15257932995@163.com (W.H.); silence@hbust.edu.cn (J.Z.); chen_hongguo1969@163.com (H.C.); caixuan@hbust.edu.cn (X.C.); yj_anna@hbust.edu.cn (J.Y.); lizeqing@hbust.edu.cn (Z.L.); cengxiangling@hbust.edu.cn (X.Z.); wuyh0914@163.com (Y.W.); 2 State Key Laboratory of Woody Oil Resources Utilization, College of Forestry, Central South University of Forestry and Technology, Changsha 410018, China; 3 State Key Laboratory of Tree Genetics and Breeding, College of Forestry and Grassland, Nanjing Forestry University, Nanjing 210037, China

**Keywords:** *Osmanthus fragrans*, low-temperature stress, melatonin, brassinolide, transcriptome, metabolome

## Abstract

Low-temperature stress severely restricts the growth, development, and ornamental value of *Osmanthus fragrans* Lour. However, the molecular mechanisms by which brassinolide (BR) and melatonin (MT) alleviate low-temperature-induced damage remain unclear. Here, *O. fragrans* branches were exposed to low-temperature stress (5, 0, −5, −10, −15, and −20 °C for 12 h) and treated with exogenous MT (50, 100, and 200 μM) or BR (0.5, 1, and 2 μM). An integrated approach combining phenotypic observation, physiological measurements, transcriptomics, and metabolomics was employed to elucidate the regulatory mechanisms underlying BR- and MT-mediated cold tolerance. The results showed that low-temperature stress significantly increased electrolyte leakage (EL), malondialdehyde (MDA), and hydrogen peroxide (H_2_O_2_) accumulation, while reducing superoxide dismutase (SOD), peroxidase (POD), and catalase (CAT) activities. Compared with the control, BR and MT treatments alleviated leaf chlorosis and wilting, reduced oxidative damage, and enhanced antioxidant enzyme activities. Integrated transcriptome–metabolome analyses demonstrated that BR and MT commonly activated phenylpropanoid and flavonoid biosynthesis, thereby promoting antioxidant metabolite accumulation, while suppressing *α*-linolenic acid and linoleic acid metabolism associated with stress-induced lipid remodeling. Network-based transcriptomic analyses identified transcription factors, including *ARF*, *EIL*, *bHLH*, and *GRAS*, as potential regulators of cold-responsive pathways. Furthermore, BR primarily regulated hormone-responsive networks, whereas MT mainly maintained redox homeostasis and metabolic reprogramming. These findings reveal the coordinated regulatory mechanisms underlying BR- and MT-mediated cold tolerance, providing potential targets for improving cold resilience in *O. fragrans*.

## 1. Introduction

Cold stress represents one of the most detrimental abiotic stresses limiting plant growth, development, geographical distribution, and ornamental performance [1]. Exposure to low temperatures causes membrane instability, impaired photosynthesis, excessive reactive oxygen species (ROS) accumulation, and oxidative damage, ultimately affecting plant survival and productivity [2,3,4,5,6,7,8]. To cope with cold stress, plants activate complex antioxidant defense systems involving enzymatic antioxidants, including superoxide dismutase (SOD), peroxidase (POD), catalase (CAT), and ascorbate peroxidase (APX), which play essential roles in ROS scavenging and maintaining cellular redox homeostasis, while soluble sugars, proline, and protective proteins contribute to osmotic regulation and cellular stabilization [9,10,11,12,13]. Nevertheless, the molecular basis of cold tolerance remains highly complex, particularly in perennial woody ornamental species.

Sweet osmanthus (*Osmanthus fragrans* Lour.), an evergreen woody species of the Oleaceae family native to China, is highly valued for its ornamental, aromatic, medicinal, and ecological properties, and has been widely cultivated in landscape applications and the fragrance industry [14,15,16]. However, as a typical subtropical woody species, *O. fragrans* exhibits limited cold tolerance and is highly vulnerable to low-temperature stress. Exposure to winter cold waves or prolonged chilling conditions frequently causes leaf chlorosis, membrane instability, impaired photosynthetic performance, and cold-induced injury, thereby restricting its geographical distribution, cultivation, and commercial utilization in northern China [17]. Furthermore, under the ongoing scenario of global climate change, the increasing occurrence of extreme low-temperature events poses a growing threat to the sustainable development of the *O. fragrans* industry. Therefore, a comprehensive understanding of the molecular mechanisms involved in cold stress perception, signal transduction, and tolerance regulation is essential for improving cold resilience and facilitating the genetic improvement of *O. fragrans*.

In recent years, exogenous plant growth regulators have attracted increasing attention for their potential roles in enhancing plant stress tolerance. Melatonin (MT), an indoleamine molecule widely present in plants, functions not only as a regulator of plant growth and development but also as an important signaling molecule involved in abiotic stress responses [18,19]. Increasing evidence indicates that MT enhances plant tolerance to environmental stresses by regulating ROS homeostasis, osmotic adjustment, photosynthetic processes, and stress-responsive metabolism [20,21]. Furthermore, MT modulates hormone signaling pathways, transcription factor (TF) expression, and energy metabolism, thereby promoting plant adaptation to adverse conditions [22,23]. Recent studies have demonstrated that exogenous MT application effectively alleviates cold-induced physiological damage in plants [23,24]. For instance, exogenous application of 100 μM MT enhanced chilling tolerance in peach [*Prunus persica* (L.) Batsch] fruit by inducing the expression of arginine decarboxylase (*ADC*), ornithine decarboxylase (*ODC*), and glutamate decarboxylase (*GAD*), thereby promoting polyamine and γ-aminobutyric acid (*GABA*) accumulation and reducing postharvest chilling injury [25]. These findings suggest that MT-mediated cold tolerance involves coordinated regulation of antioxidant metabolism, osmotic adjustment, and stress signaling.

Brassinolides (BRs) are important steroid hormones that regulate plant growth, development, and stress responses [26,27,28]. Increasing evidence indicates that BRs enhance cold tolerance through complex regulatory networks involving BR signaling, hormone interactions, and metabolic regulation [29,30]. BR signaling exhibits extensive crosstalk with other phytohormones, including abscisic acid (ABA), jasmonic acid (JA), and ethylene (ETH), contributing to coordinated stress adaptation [27,31]. For example, BR treatment in cucumber (*Cucumis sativus* L. var. *sativus*) seedlings activated BR-responsive pathways, modulated endogenous hormone homeostasis, enhanced antioxidant capacity, and maintained photosynthetic performance under cold stress [31]. However, the regulatory mechanisms and optimal application strategies of BR and MT vary among plant species, and their roles in cold tolerance of *O. fragrans* remain largely unknown. Therefore, elucidating BR- and MT-mediated cold stress responses in *O. fragrans* is essential for understanding its adaptive mechanisms and facilitating cold-resistant germplasm improvement and molecular breeding.

With the rapid development of high-throughput sequencing technologies, transcriptomics and metabolomics have become powerful approaches for elucidating plant responses to abiotic stresses [32,33]. Transcriptomic analysis enables the systematic identification of differentially expressed genes (DEGs) and the dynamic regulation of key signaling pathways under low-temperature conditions, whereas metabolomic analysis reflects changes in metabolite accumulation and metabolic reprogramming in response to stress. Integrated transcriptomic and metabolomic analyses not only facilitate the interpretation of plant stress adaptation at the gene–metabolite level but also enable the identification of key regulatory genes, metabolic pathways, and signaling networks [34,35]. Therefore, physiological measurements alone are insufficient to fully characterize the complex regulatory mechanisms governing cold tolerance. Multi-omics strategies, particularly combined transcriptome–metabolome analyses, offer an effective framework for unraveling the coordinated transcriptional and metabolic regulation of plant responses to low-temperature stress. Such approaches are especially valuable for dissecting the complex regulatory networks mediated by exogenous signaling molecules, including BR and MT, in non-model woody species.

Based on these considerations, *O. fragrans* was selected as an important woody ornamental species to investigate the molecular mechanisms underlying BR- and MT-mediated cold tolerance. In this study, plants were subjected to low-temperature stress combined with exogenous MT and BR treatments, followed by comprehensive phenotypic evaluation, physiological analyses, transcriptomic profiling, and metabolomic characterization to assess their roles in alleviating cold-induced damage. We hypothesized that BR and MT enhance cold adaptation through distinct yet interconnected regulatory networks involving antioxidant defense, transcriptional regulation, and metabolic reprogramming. This study aims to elucidate the protective mechanisms of BR and MT under low-temperature stress and identify potential regulatory factors associated with cold tolerance. This study provides new insights into the distinct and convergent regulatory mechanisms of BR and MT in enhancing cold tolerance in *O. fragrans* and provides valuable molecular resources for cold-resistant breeding and sustainable landscape applications of this economically important woody ornamental species.

## 2. Results

### 2.1. Phenotypic Analysis

**Figure 1:**
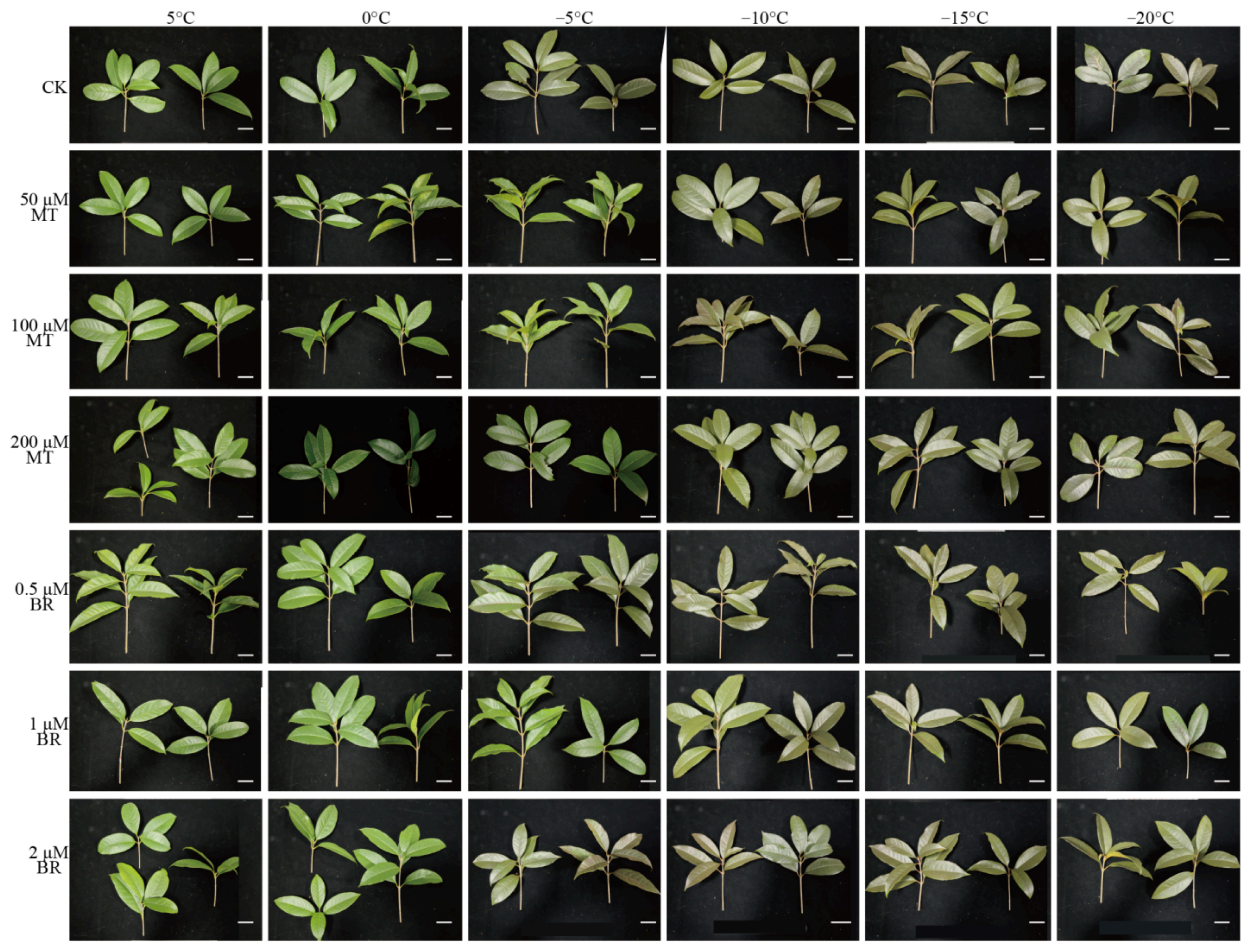
Effects of different concentrations of melatonin (MT) and brassinolide (BR) treatments on the phenotypic responses of *Osmanthus fragrans* Lour. leaves under low-temperature stress. CK indicates H_2_O-treated control. MT was applied at 50, 100, and 200 μM, while BR was applied at 0.5, 1, and 2 μM. After treatment, leaves were exposed to low-temperature conditions at 5, 0, −5, −10, −15, and −20 °C, respectively. Bars = 3 cm.

With decreasing temperature from 5 to −20 °C, cold-induced damage in *O. fragrans* leaves progressively increased, as evidenced by chlorosis, wilting, and leaf curling (Figure 1). H_2_O-treated control (CK) plants maintained normal morphology and green coloration at 5 and 0 °C. However, visible dehydration and chlorosis appeared when the temperature dropped below −5 °C, and severe phenotypic damage occurred at −20 °C. Compared with CK plants, exogenous MT and BR treatments attenuated low-temperature-induced damage to different extents. Under moderate cold stress (−5 °C), both treatments largely preserved leaf greenness and structural integrity, whereas under severe cold stress (−10 to −20 °C), MT- and BR-treated plants exhibited markedly reduced wilting and chlorosis (Figure 1). Among the tested concentrations, 100 μM MT and 1 μM BR exhibited the most pronounced protective effects, whereas other concentrations (50 and 200 μM MT; 0.5 and 2 μM BR) showed relatively weaker protection (Figure 1).

**Figure 2:**
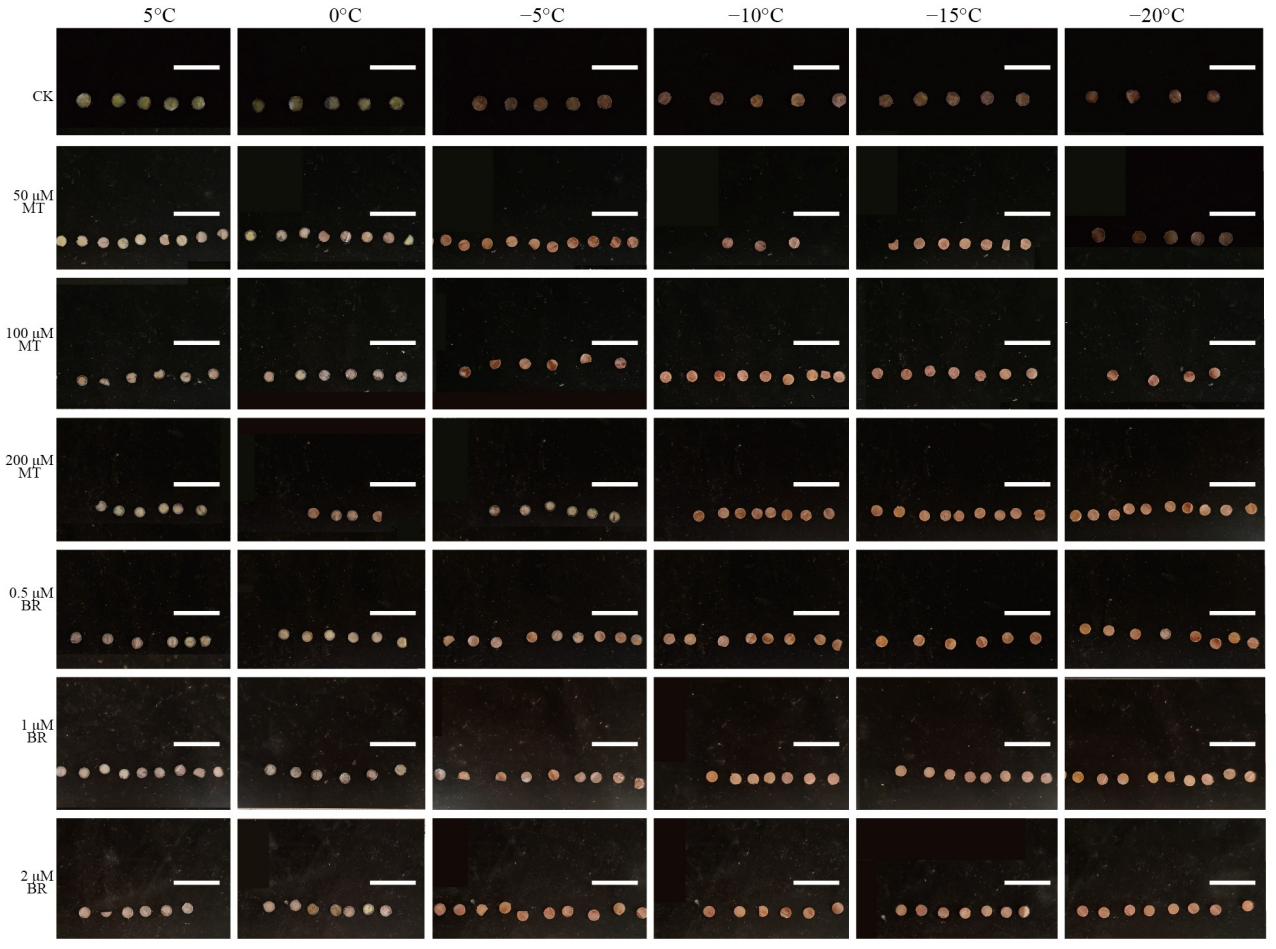
NBT staining of leaves under low-temperature stress. CK indicates water-treated control. MT was applied at 50, 100, and 200 μM, while BR was applied at 0.5, 1, and 2 μM. After treatment, leaves were exposed to low-temperature conditions at 5, 0, −5, −10, −15, and −20 °C. Bars = 3 cm.

**Figure 3:**
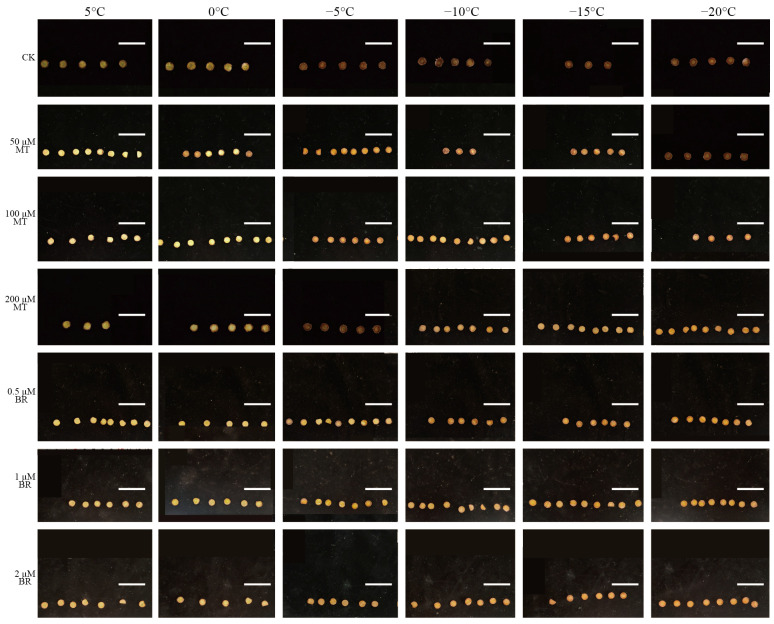
DAB staining of leaves under low-temperature stress. CK indicates water-treated control. MT was applied at 50, 100, and 200 μM, while BR was applied at 0.5, 1, and 2 μM. After treatment, leaves were exposed to low-temperature conditions at 5, 0, −5, −10, −15, and −20 °C. Bars = 3 cm.

Nitroblue tetrazolium (NBT) and 3,3′-diaminobenzidine (DAB) staining further demonstrated that low-temperature stress markedly induced ROS accumulation in *O. fragrans* leaves (Figure 2 and Figure 3). With decreasing temperature, the staining intensity of both NBT and DAB progressively increased (Figure 2 and Figure 3), suggesting enhanced accumulation of O_2_^−^ and hydrogen peroxide (H_2_O_2_). In CK plants, NBT and DAB staining intensity increased markedly below −5 °C and reached the highest levels at −20 °C. In contrast, exogenous MT and BR treatments markedly reduced NBT and DAB staining intensity under different temperature conditions. Notably, 100 μM MT and 1 μM BR consistently exhibited the weakest staining signals across all temperature treatments (Figure 2 and Figure 3). These results indicate that MT and BR treatments effectively reduced ROS accumulation and alleviated oxidative stress under low-temperature conditions, which may contribute to enhanced cold tolerance in *O. fragrans*.

### 2.2. Physiological Responses

**Figure 4:**
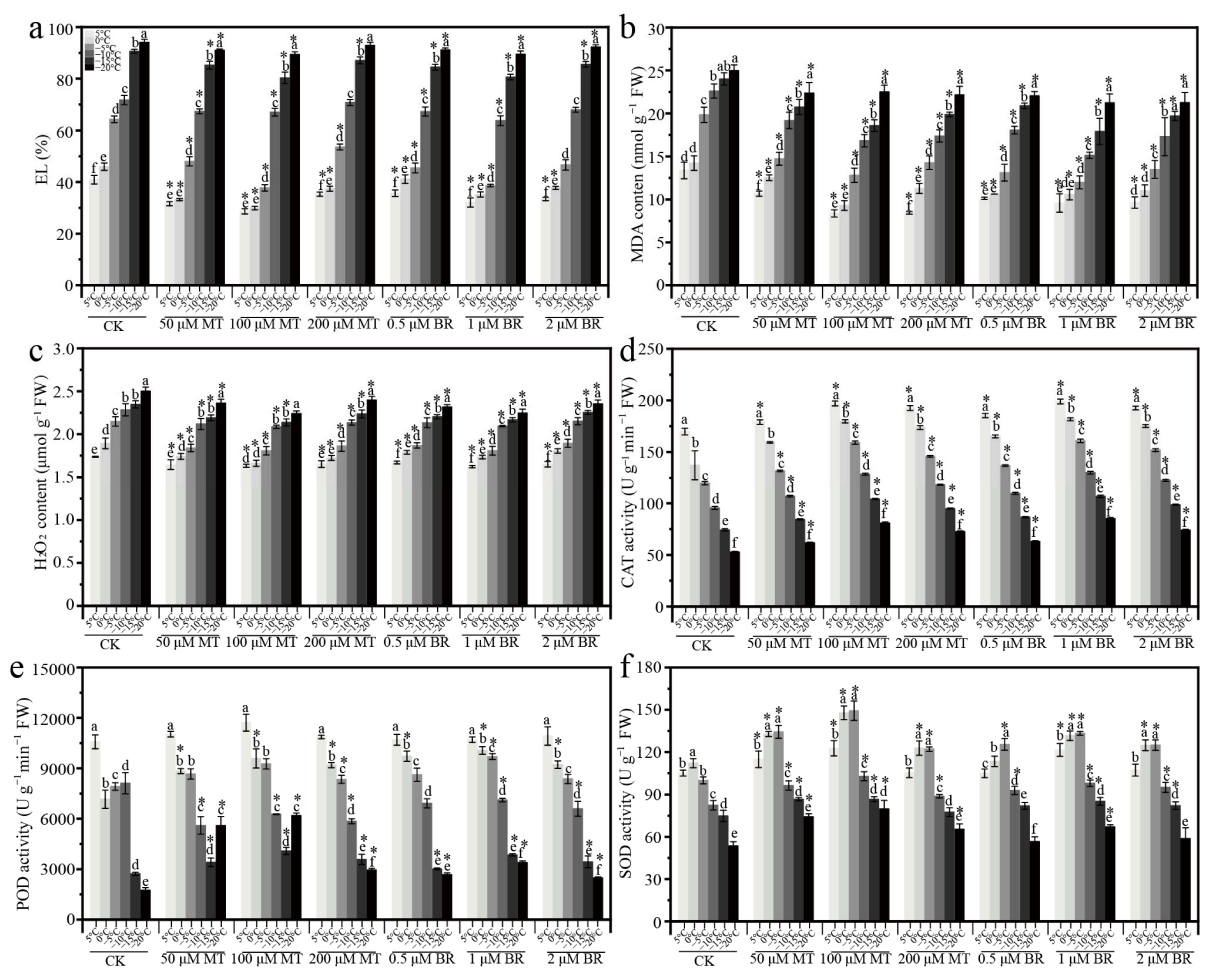
Physiological parameters. (**a**) Electrolyte leakage (EL); (**b**) malondialdehyde (MDA) content; (**c**) hydrogen peroxide (H_2_O_2_) content; (**d**) catalase (CAT) activity; (**e**) peroxidase (POD) activity; and (**f**) superoxide dismutase (SOD) activity. Data are presented as mean ± standard deviation (SD). Different lowercase letters indicate statistically significant differences among different temperature treatments within the same treatment, while asterisks (*) indicate significant differences between each treatment and the corresponding CK within the same temperature condition.

With decreasing temperature from 5 to −20 °C, indicators associated with membrane damage and oxidative stress progressively increased in *O. fragrans* leaves (Figure 4a–c). In CK plants, electrolyte leakage (EL) increased significantly (*p* < 0.05) with decreasing temperature, rising from 40.88% at 5 °C to 94.07% at −20 °C (Figure 4a). Meanwhile, malondialdehyde (MDA) and H_2_O_2_ contents exhibited continuous and significant (*p* < 0.05) increases, rising from approximately 13.37 nmol g^−1^ FW and 1.74 mmol g^−1^ FW to 24.98 nmol g^−1^ FW and 2.50 mmol g^−1^ FW, respectively (Figure 4b,c). Compared with CK plants, exogenous MT and BR treatments significantly (*p* < 0.05) reduced EL, MDA, and H_2_O_2_ accumulation under low-temperature stress. Notably, 100 μM MT showed the strongest mitigating effect, reducing EL by 41.28% at −5 °C compared with CK plants and maintaining lower MDA and H_2_O_2_ accumulation across temperature treatments. Similarly, 1 μM BR exhibited stronger protective effects than 0.5 μM and 2 μM BR treatments (Figure 4a–c).

Low-temperature stress markedly reduced antioxidant enzyme activities in *O. fragrans* leaves (Figure 4d–f). In CK plants, the activities of CAT, POD, and SOD progressively declined with decreasing temperature, reaching 52.67 U g^−1^ min^−1^ FW, 1761.11 U g^−1^ min^−1^ FW, and 53.48 U g^−1^ FW at −20 °C, respectively (Figure 4d–f). In contrast, MT and BR treatments significantly (*p* < 0.05) enhanced antioxidant enzyme activities under cold stress. Notably, 100 μM MT sustained higher CAT, POD, and SOD activities across all temperature treatments, while 1 μM BR exhibited a similar enhancing effect and outperformed both 0.5 μM and 2 μM BR treatments (Figure 4d–f). Overall, optimal concentrations of MT and BR enhanced antioxidant defense capacity and reduced oxidative damage associated with membrane lipid peroxidation, thereby contributing to improved cold tolerance in *O. fragrans*.

### 2.3. Transcriptomic Analysis

Based on the preliminary physiological screening, 100 μM MT and 1 μM BR were identified as the optimal treatments for improving cold tolerance and were selected for subsequent transcriptomic analysis. To comprehensively characterize the transcriptional responses of *O. fragrans* to cold stress and exogenous MT/BR treatments, four representative temperature conditions (25, 5, −5, and −10 °C) were selected for RNA-seq analysis. A total of 1,253,953,870 raw reads were generated from 30 RNA-seq libraries (Table S1). After quality control (QC), including adapter removal and filtering of low-quality reads, 1,253,303,052 clean reads were obtained, with clean read ratios exceeding 99.45% across all libraries (Table S1). The sequencing datasets exhibited excellent quality, with Q20 values above 98.68%, Q30 values above 96.70%, and GC contents above 39.95% (Table S1). Furthermore, 75.07–91.97% of clean reads were successfully mapped to the reference genome (Table S2), indicating the reliability of the RNA-seq datasets for subsequent analyses.

To further assess dataset reproducibility and sample consistency, gene expression distribution, principal component analysis (PCA), genomic feature distribution, and sample correlation analyses were performed (Figure S1). Boxplot analysis of log_2_(FPKM + 1) values showed highly similar expression distributions across samples, with comparable medians and interquartile ranges, indicating consistent sequencing performance and strong sample comparability (Figure S1a). PCA revealed that the first two principal components (PC1 and PC2) explained 35.61% and 16.46% of the total variance, respectively. Biological replicates clustered closely within each treatment group, while distinct separation among treatments was observed, indicating treatment-associated variation in global gene expression patterns (Figure S1b). Genomic feature annotation revealed that most reads were mapped to coding sequences (CDS), followed by 3′ untranslated regions (3′UTRs), intronic regions, and intergenic regions, with highly similar distributions across samples (Figure S1c). Correlation analysis further demonstrated high reproducibility among biological replicates, whereas lower correlations were observed between different treatment groups. Hierarchical clustering showed that samples were primarily grouped according to treatment conditions (Figure S1d), supporting the robustness of the experimental design and the suitability of the datasets for downstream differential expression analysis.

Across the 15 comparison groups, a total of 20,149 non-redundant DEGs were identified (Table S3). Under identical temperature conditions, exogenous BR and MT treatments induced substantial transcriptional alterations. Notably, at −5 °C, 7754 and 10,462 DEGs were detected in the BR- and MT-treated groups, respectively, representing stronger transcriptional responses than those observed at 5 °C and −10 °C (Table S3). In CK plants, temperature changes alone also resulted in extensive transcriptomic variation, with 13,108, 10,491, and 4780 DEGs detected in the −10H_2_O_vs_5H_2_O, −5H_2_O_vs_5H_2_O, and −10H_2_O_vs_−5H_2_O comparisons, respectively (Table S3). Similar patterns of transcriptional reprogramming were observed in BR- and MT-treated plants across different temperature contrasts. The highest number of DEGs was detected in the −10MT_vs_−5MT comparison (13,762 DEGs), followed by −10BR_vs_−5BR (11,450 DEGs) and −10MT_vs_5MT (10,773 DEGs) (Table S3). Overall, these results indicate that temperature changes triggered extensive transcriptomic remodeling, while exogenous BR and MT treatments further reshaped cold-responsive gene expression profiles in *O. fragrans*.

### 2.4. Weighted Gene Coexpression Network Analysis (WGCNA)

**Figure 5:**
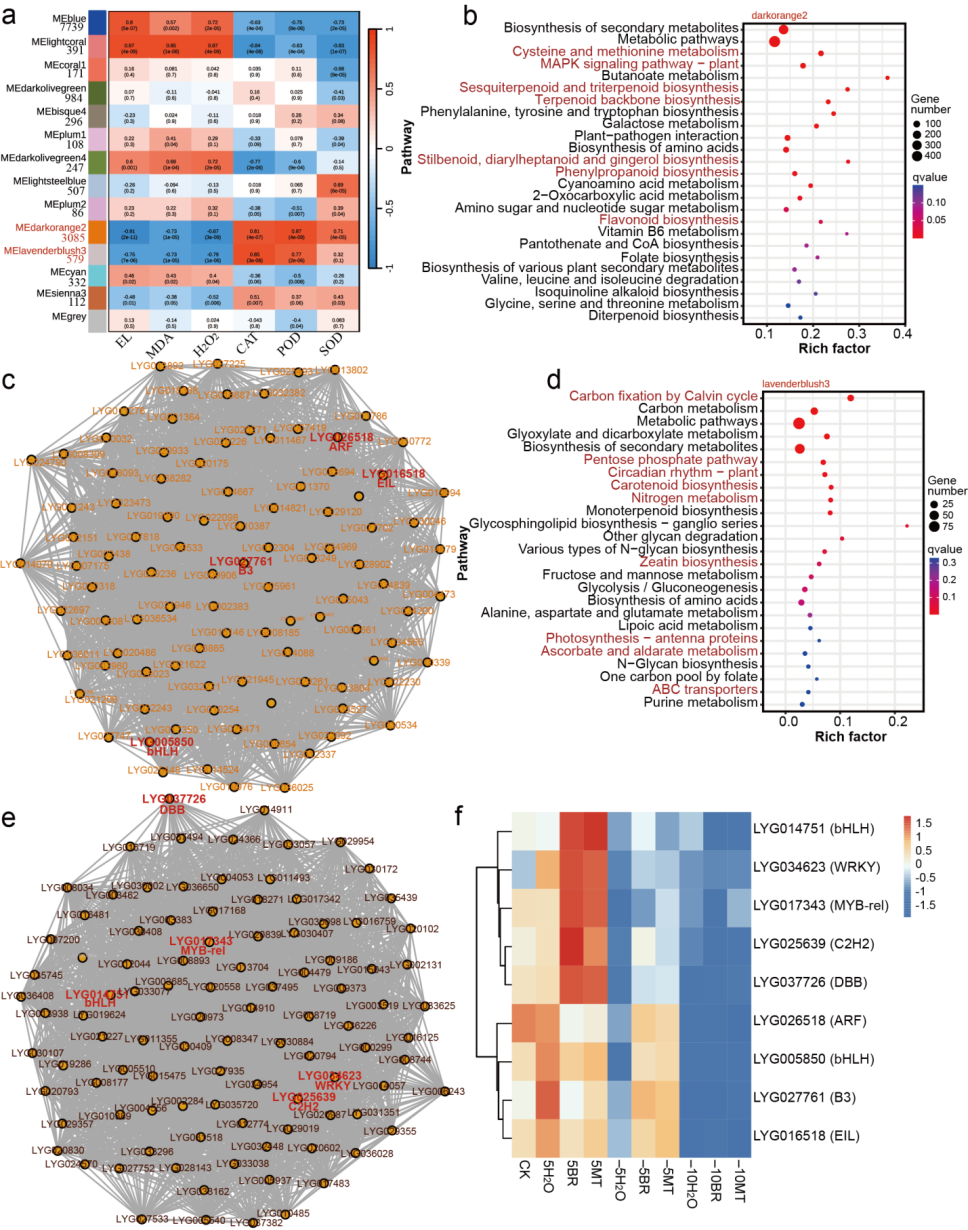
Weighted gene coexpression network analysis (WGCNA) based on differentially expressed genes (DEGs) across all comparison groups. (**a**) Module–trait correlation heatmap. (**b**) Enrichment analysis of DEGs in the darkorange2 module. (**c**) Network diagrams of the top 100 hub genes in the darkorange2 module. (**d**) Enrichment analysis of DEGs in the lavenderblush3 module. (**e**) Network diagrams of the top 100 hub genes in the lavenderblush3 module. (**f**) Hierarchical clustering heatmap of key TFs.

WGCNA was performed using 20,149 DEGs integrated with physiological trait data. Genes exhibiting low expression variability (standard deviation (SD) ≤ 0.5) were removed, yielding 14,939 genes that were clustered into 14 coexpression modules (Figure 5a). Among these modules, the blue module contained the largest number of genes (7739), followed by the darkorange2 module (3085 genes) (Figure 5a).

Module–trait correlation analysis revealed that the darkorange2 and lavenderblush3 modules were significantly negatively correlated with EL, MDA, and H_2_O_2_ accumulation, while positively correlated with antioxidant enzyme activities, particularly CAT and SOD (Figure 5a). Functional enrichment analysis showed that genes in the darkorange2 module were significantly enriched in stress-related pathways, including plant MAPK signaling, cysteine and methionine metabolism, phenylpropanoid biosynthesis, flavonoid biosynthesis, and terpenoid metabolism (Figure 5b). Several key TFs identified in this module included *LYG026518* (*ARF*), *LYG016518* (*EIL*), *LYG027761* (*B3*), and *LYG005850* (*bHLH*) (Figure 5c). In contrast, the lavenderblush3 module was mainly associated with primary metabolism, photosynthetic maintenance, and stress adaptation, with significant enrichment in pathways such as carbon fixation in the Calvin cycle, pentose phosphate pathway, circadian rhythm, carotenoid biosynthesis, nitrogen metabolism, zeatin biosynthesis, photosynthesis-antenna proteins, ascorbate and aldarate metabolism, and ABC transporters (Figure 5d). Representative TFs in this module included *LYG034623* (*WRKY*), *LYG025639* (*C2H2*), *LYG014751* (*bHLH*), *LYG017343* (*MYB-related*), and *LYG037726* (*DBB*) (Figure 5e). Hierarchical clustering analysis further revealed distinct expression patterns of hub TFs among treatments. In the darkorange2 module, most hub TFs showed reduced expression at 5 °C but were markedly induced under −5 °C conditions following BR and MT treatments compared with CK plants (Figure 5f). Conversely, TFs in the lavenderblush3 module generally exhibited increased expression under both 5 °C and −5 °C conditions in BR- and MT-treated plants relative to CK plants (Figure 5f). Collectively, these results suggest that the darkorange2 and lavenderblush3 modules are associated with BR- and MT-mediated cold responses by integrating antioxidant defense, stress signaling, secondary metabolism, photosynthetic regulation, and hormone-related pathways in *O. fragrans*.

### 2.5. Multi-Layer Gene Regulatory Network Analysis to Identify Key Regulatory Genes

**Figure 6:**
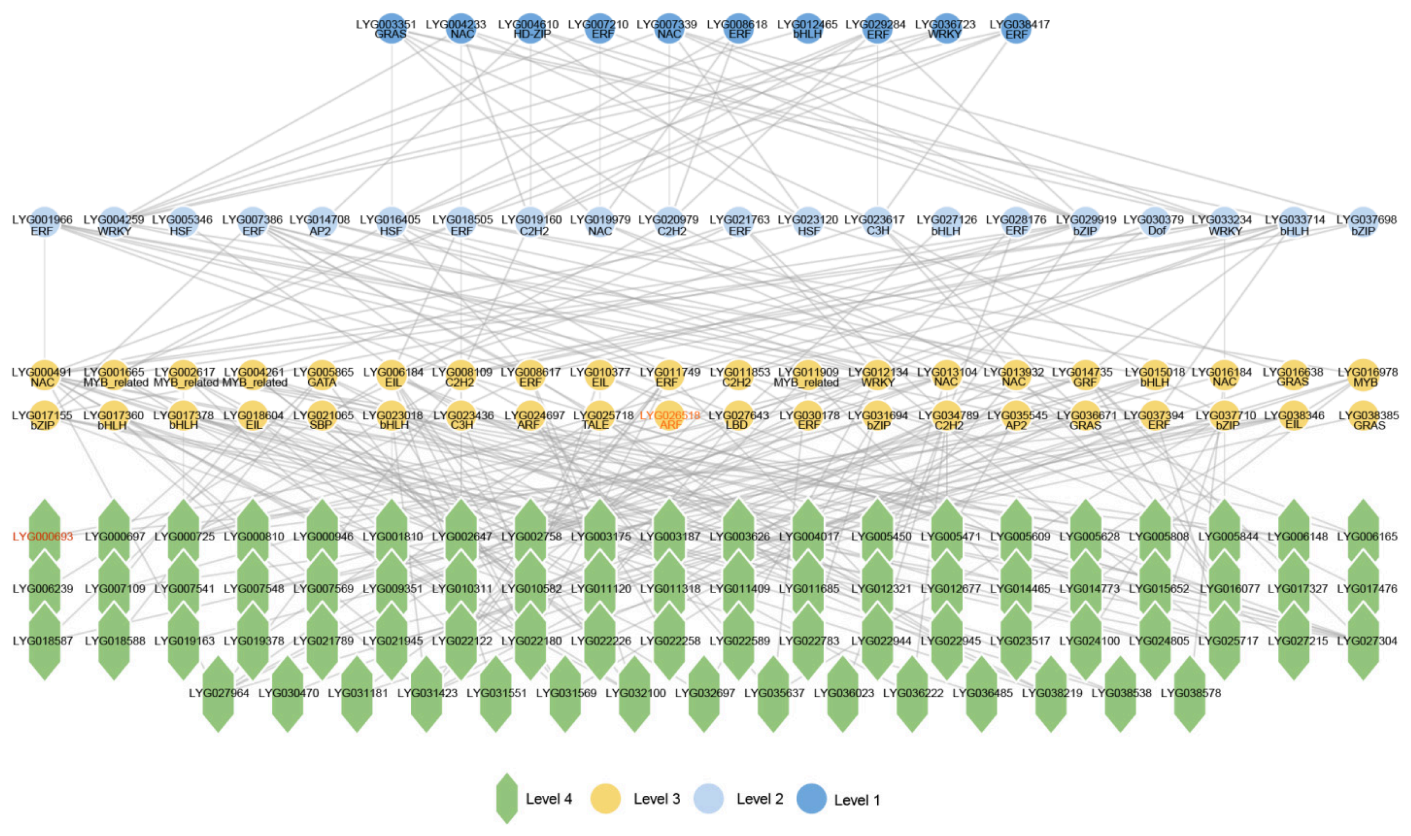
ML-hGRN of the low-temperature stress response in *O. fragrans*. The inferred ML-hGRN consists of three TF layers (levels 1–3, each containing TFs) and one downstream structural gene layer (level 4).

To further investigate the hierarchical transcriptional regulation underlying BR- and MT-mediated cold responses, a multi-layer hierarchical gene regulatory network (ML-hGRN) was constructed using the BWERF algorithm (Figure 6). The predicted network was organized into four hierarchical layers, with Layer 1 containing 10 TFs representing potential upstream regulators. Layers 2 and 3 comprised 20 and 40 TFs, respectively, while the bottom layer consisted of 75 structural genes. In total, 290 regulatory interactions were predicted, including 42 interactions between Layers 1 and 2, 70 interactions between Layers 2 and 3, and 178 interactions between Layer 3 TFs and structural genes (Figure 6). These results indicate a hierarchical regulatory organization, in which upstream TFs were predicted to modulate downstream effector genes involved in cold stress responses.

To further characterize the dynamic expression patterns of regulatory factors within the ML-hGRN, the expression profiles of TFs across the first three layers were analyzed (Table S4). Most TFs in Layers 1 and 2 were downregulated under BR and MT treatments compared with the corresponding CK samples at 5, −5, and −10 °C, with the exception of *LYG014708* (*AP2*), which exhibited a distinct expression pattern (Table S4). In contrast, Layer 3 TFs exhibited greater expression diversity, with both upregulation and downregulation observed under different treatment conditions. Notably, several TF families, including *MYB-related* (*LYG001665* and *LYG004261*), *EIL* (*LYG018604*, *LYG038346*, and *LYG010377*), *SBP* (*LYG021065*), *bHLH* (*LYG023018* and *LYG017360*), *C3H* (*LYG023436*), *ARF* (*LYG024697* and *LYG026518*), *TALE* (*LYG025718*), *bZIP* (*LYG031694* and *LYG017155*), *C2H2* (*LYG034789*), *AP2* (*LYG035545*), *GRAS* (*LYG038385* and *LYG016638*), *GATA* (*LYG005865*), and *GRF* (*LYG014735*), exhibited relatively consistent upregulation trends under BR and MT treatments at 5 °C and −5 °C compared with CK plants (Table S4).

Among these regulators, members of the *EIL*, *ARF*, *bHLH*, *bZIP*, and *GRAS* families showed high connectivity within the network and exhibited strong induction trends under BR and MT treatments (Figure 6). Notably, *LYG026518* (*ARF*) displayed the highest connectivity among the identified TFs and was predicted as a potential hub TF, suggesting its possible involvement in BR- and MT-mediated transcriptional regulation during low-temperature stress responses (Figure 6). Overall, the ML-hGRN revealed a hierarchical transcriptional regulatory framework, in which upstream TFs may modulate downstream structural genes through multi-level regulatory interactions, thereby contributing to adaptive responses to low-temperature stress in *O. fragrans*.

### 2.6. Metabolomic Analysis

A total of 2936 metabolites were detected and annotated across the 30 samples, including 1482 metabolites identified in positive ion mode and 1454 in negative ion mode (Table S5). In positive ion mode, the major metabolite classes were others (18.65%), benzenoids (16.12%), organic acids and derivatives (10.86%), alkaloids and derivatives (8.80%), and lipids and lipid-like molecules (8.15%). In negative ion mode, the predominant classes included others (20.06%), organic acids and derivatives (19.81%), benzenoids (13.71%), and flavonoids (11.03%) (Figure S2).

To evaluate data quality and analytical stability, QC analyses were performed (Figure S3). In both positive and negative ion modes, total ion chromatograms (TICs) of QC samples showed strong overlap, with highly consistent retention times and peak intensities, indicating stable instrument performance and good reproducibility (Figure S3a,b). Correlation analysis further demonstrated high consistency among QC samples, with correlation coefficients ranging from 0.9993 to 0.9997 in positive ion mode and from 0.9999 to 1.0000 in negative ion mode (Figure S3c,d). PCA showed that the first two components (PC1 and PC2) explained 50.60% and 8.36% of the total variance, respectively (Figure S4). Biological replicates clustered closely within each treatment group, whereas distinct separation among treatment conditions was observed (Figure S4), indicating strong metabolic consistency within groups and treatment-associated metabolic variation among groups.

Across the 15 pairwise comparisons, 184–848 differentially accumulated metabolites (DAMs) were identified (Table S6). Under the same temperature conditions, both BR and MT treatments induced substantial metabolic alterations compared with CK plants. At −5 °C, 721 and 718 DAMs were detected in the BR and MT-treated groups, respectively, representing more pronounced metabolic alterations than those observed at 5 °C (184 and 205 DAMs, respectively). At −10 °C, BR and MT treatments resulted in 486 and 567 DAMs, respectively (Table S6). In temperature-shift comparisons within the H_2_O-treated CK group, 481–779 DAMs were identified across different temperature contrasts. Similarly, BR- and MT-treated plants exhibited extensive metabolic changes under different temperature transitions. Among all comparisons, the –10MT_vs_5MT comparison showed the highest number of DAMs (848), whereas the –5BR_vs_5BR comparison showed the lowest number (203) (Table S6).

### 2.7. Integrated Transcriptome and Metabolome Analysisto Identify Key Pathways

**Figure 7:**
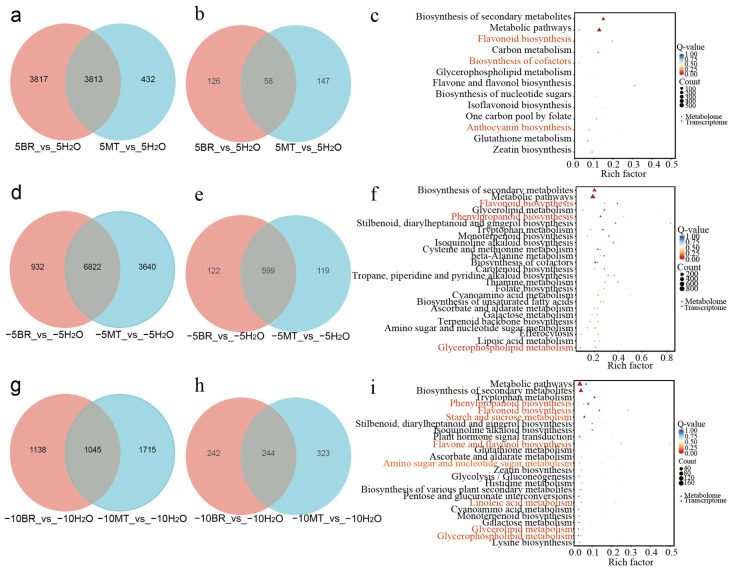
DEGs and differentially accumulated metabolites (DAMs) analysis under different treatments at the same temperature. DEGs (**a**) and DAMs (**b**) at 5 °C. (**c**) Joint enrichment analysis of DEGs and DAMs at 5 °C. DEGs (**d**) and DAMs (**e**) at −5 °C. (**f**) Joint enrichment analysis of DEGs and DAMs at −5 °C. DEGs (**g**) and DAMs (**h**) at −10 °C. (**i**) Joint enrichment analysis of DEGs and DAMs at −10 °C. Squares represent DAMS, and triangles represent DEGs.

To elucidate the molecular mechanisms underlying BR and MT-mediated cold responses, an integrated analysis of DEGs and DAMs was conducted under different temperature conditions. At 5 °C, 3813 DEGs and 58 DAMs were identified in the 5BR_vs_5H_2_O and 5MT_vs_5H_2_O comparisons, respectively (Figure 7a,b). Kyoto Encyclopedia of Genes and Genomes (KEGG)-based joint enrichment analysis revealed that these DEGs and DAMs were mainly associated with flavonoid biosynthesis, anthocyanin biosynthesis, and cofactor biosynthesis (Figure 7c). At −5 °C, 6822 DEGs and 599 DAMs were identified in the −5BR_vs_−5H_2_O and −5MT_vs_−5H_2_O comparisons, respectively (Figure 7d,e). Joint enrichment analysis showed that flavonoid biosynthesis, phenylpropanoid biosynthesis, and glycerophospholipid metabolism were among the most significantly enriched pathways (Figure 7f). At −10 °C, 1045 DEGs and 244 DAMs were detected in the −10BR_vs_−10H_2_O and −10MT_vs_−10H_2_O comparisons, respectively (Figure 7g,h). These DEGs and DAMs were significantly enriched in pathways related to phenylpropanoid biosynthesis, flavonoid biosynthesis, starch and sucrose metabolism, flavone and flavonol biosynthesis, amino sugar and nucleotide sugar metabolism, linoleic acid metabolism, glycerolipid metabolism, and glycerophospholipid metabolism (Figure 7i). These results indicate that BR and MT treatments modulate cold-responsive processes in *O. fragrans* through coordinated regulation of secondary metabolism, carbohydrate metabolism, and lipid metabolism, particularly involving phenylpropanoid and flavonoid biosynthesis pathways.

**Figure 8:**
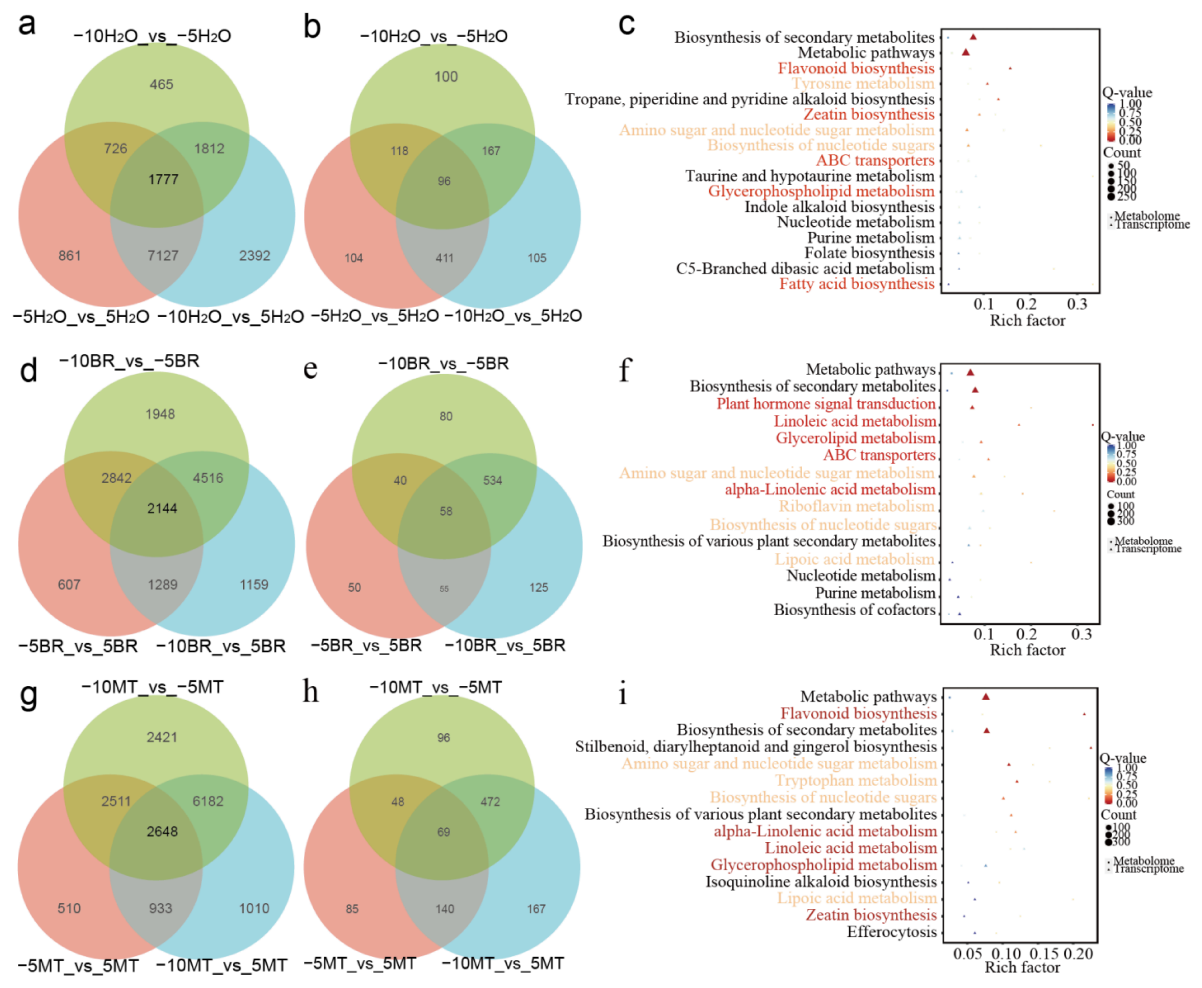
DEGs and DAMs analysis under different temperature treatments with the same treatment. H_2_O treatment: DEGs (**a**) and DAMs (**b**) analysis; (**c**) joint enrichment analysis of DEGs and DAMs under H_2_O treatment. BR treatment: DEGs (**d**) and DAMs (**e**) analysis; (**f**) joint enrichment analysis of DEGs and DAMs under BR treatment. MT treatment: DEGs (**g**) and DAMs (**h**) analysis; (**i**) joint enrichment analysis of DEGs and DAMs at −10 °C. Squares represent DAMS, and triangles represent DEGs.

To further explore shared regulatory responses across temperature gradients, common DEGs and DAMs among different temperature comparisons were analyzed (Figure 8). In the CK group, comparisons among −10H_2_O_vs_−5H_2_O, −5H_2_O_vs_5H_2_O, and −10H_2_O_vs_5H_2_O identified 1777 shared DEGs and 95 shared DAMs (Figure 8a,b). These shared DEGs and DAMs were mainly enriched in flavonoid biosynthesis, zeatin biosynthesis, ABC transporters, glycerophospholipid metabolism, and fatty acid biosynthesis (Figure 8c). In the BR treatment group, 2144 shared DEGs and 59 shared DAMs were identified across the −10BR_vs_−5BR, −5BR_vs_5BR, and −10BR_vs_5BR comparisons (Figure 8d,e). These shared molecules were mainly associated with plant hormone signal transduction, *α*-linolenic acid metabolism, linoleic acid metabolism, glycerolipid metabolism, and ABC transporters (Figure 8f). Similarly, in the MT treatment group, 2648 shared DEGs and 69 shared DAMs were identified across the corresponding temperature comparisons (Figure 8g,h), with predominant enrichment in flavonoid biosynthesis, *α*-linolenic acid metabolism, linoleic acid metabolism, glycerophospholipid metabolism, and zeatin biosynthesis (Figure 8i). These results suggest that BR and MT may contribute to enhanced cold tolerance in *O. fragrans* through coordinated regulation of membrane lipid remodeling, hormone signaling, and antioxidant-related secondary metabolism.

### 2.8. Phenylpropanoid and Flavonoid Biosynthesis Pathways

**Figure 9:**
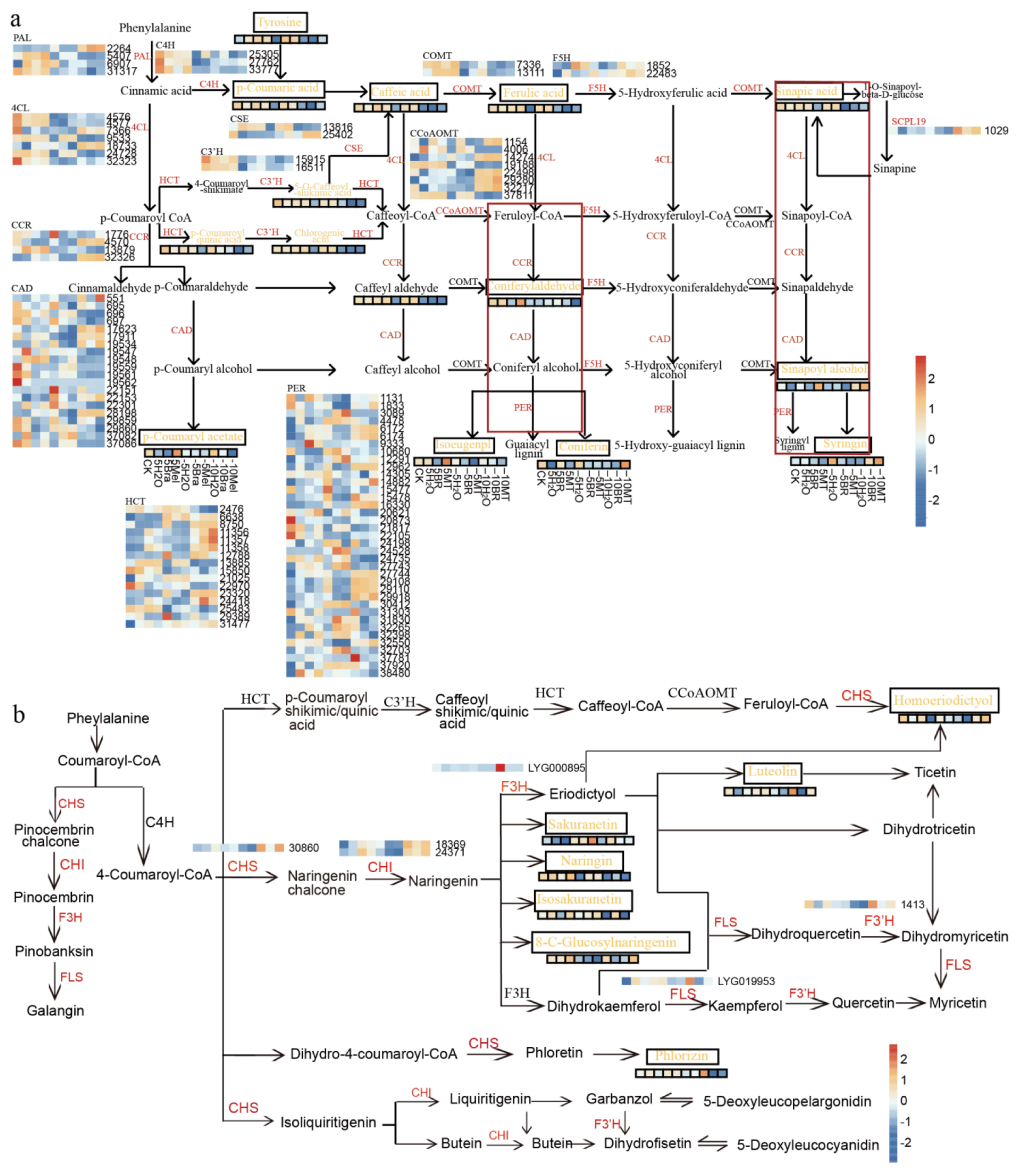
Analysis of DEGs and DAMs in the phenylpropanoid metabolic pathway and flavonoid pathways. (**a**) Phenylpropanoid pathway; and (**b**) flavonoid pathway. Heatmaps with black frames represent DAMs, while those without black frames represent DEGs.

In the phenylpropanoid biosynthesis pathway, 108 DEGs and 14 DAMs were identified (Figure 9a). Under −5 °C stress, compared with H_2_O-treated CK plants, BR and MT treatments significantly increased the accumulation of key metabolites, including L-tyrosine, *p*-coumaroyl quinic acid, 4-coumarate, 5-O-caffeoylshikimic acid, caffeate, ferulate, sinapate, and chlorogenate (Figure 9a). Among these metabolites, chlorogenate showed the most pronounced accumulation increase, with 10.11- and 10.24-fold higher accumulation under BR and MT treatments, respectively, compared with CK plants (Figure 9a). Consistently, several structural genes involved in this pathway were significantly upregulated, including *4CL*, *COMT*, *CSE*, *C4H*, *C3′H*, *CCoAOMT*, and *PRDX6* (Figure 9a). The coordinated induction of biosynthetic genes and accumulation of pathway intermediates suggests that BR and MT activate phenylpropanoid metabolism, thereby promoting phenolic compound biosynthesis and potentially contributing to improved cold tolerance in *O. fragrans*.

In the flavonoid biosynthesis pathway, 35 DEGs and 10 DAMs were identified (Figure 9b). Under −5 °C stress, compared with H_2_O-treated CK plants, BR and MT treatments significantly increased the accumulation of multiple flavonoid- and phenylpropanoid-derived metabolites (Figure 9b). Chlorogenate showed the greatest increase, with 10.24- and 10.11-fold higher accumulation under BR and MT treatments, respectively. In addition, *p*-coumaroyl quinic acid (5.90- and 2.17-fold), 5-O-caffeoylshikimic acid (4.80- and 4.74-fold), and naringin (4.72- and 4.68-fold) showed significantly increased accumulation in BR- and MT-treated samples (Figure 9b). In contrast, several flavonoid compounds, such as luteolin, exhibited reduced accumulation under low-temperature stress (Figure 9b). Meanwhile, key structural genes involved in flavonoid biosynthesis, including *C4H* and *C3′H*, were significantly upregulated in both BR- and MT-treated groups under −5 °C stress (Figure 9b). Collectively, the coordinated changes in transcript abundance and metabolite accumulation suggest that BR and MT promote phenylpropanoid-derived flavonoid biosynthesis, thereby facilitating phenolic compound production and potentially contributing to enhanced cold tolerance in *O. fragrans*.

### 2.9. α-Linolenic Acid and Linoleic Acid Metabolism

**Figure 10:**
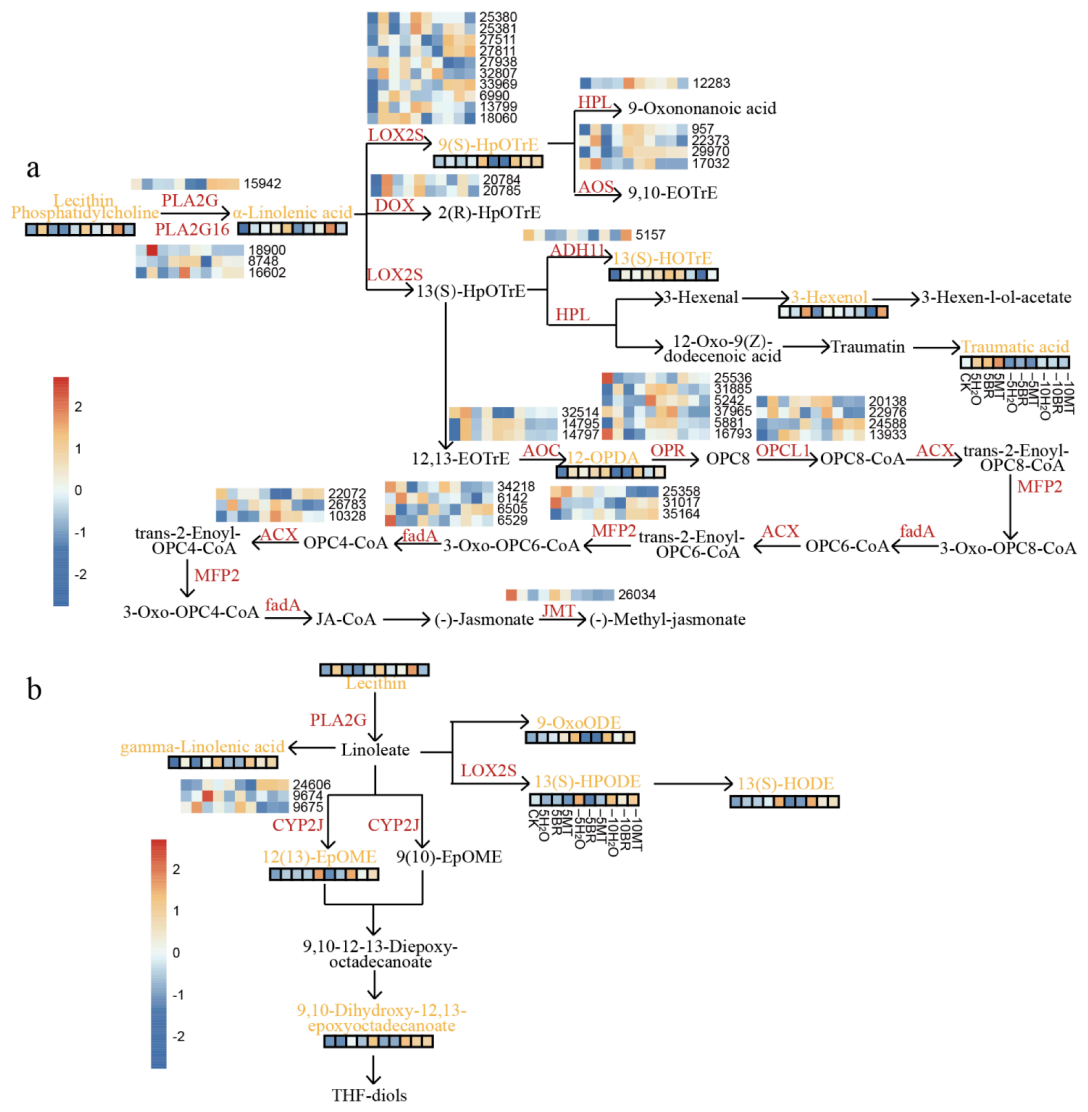
α-Linolenic acid metabolism and linoleic acid metabolism. (**a**) α-Linolenic acid metabolism; and (**b**) linoleic acid metabolism. Heatmaps with black frames represent DAMs, while those without black frames represent DEGs.

In the *α*-linolenic acid metabolism pathway, 46 DEGs corresponding to 14 genes and 7 DAMs were identified (Figure 10a). Under −5 °C stress, compared with H_2_O-treated CK plants, BR and MT treatments significantly altered the expression patterns of pathway-related genes. Ten genes, including *ADH11*, *AOC*, *AOS*, *DOX*, *JMT*, *HPL*, *LOX2S*, *MFP2*, *PLA2G*, and *PLA2G16*, exhibited overall downregulation patterns. Among them, *DOX*, *JMT*, *HPL*, *PLA2G*, and *PLA2G16* showed the most pronounced decreases, with transcript levels reduced by 57.16–96.58% relative to CK plants (Figure 10a). Correspondingly, only two key metabolites, 12-OPDA and 9(S)-HPOT, showed significant changes, with their accumulation levels decreasing by 86.69−94.52% under BR and MT treatments (Figure 10a). These results suggest that BR and MT treatments modulate *α*-linolenic acid metabolism by suppressing the accumulation of oxylipin precursors under cold stress, thereby contributing to the regulation of cold-responsive metabolic processes in *O. fragrans*.

In the linoleic acid metabolism pathway, 14 DEGs and 7 DAMs were identified (Figure 10b). Under −5 °C stress, compared with H_2_O-treated CK plants, BR and MT treatments significantly reduced the accumulation of six key metabolites, including 13-HODE, 13-HpODE, 9-OxoODE, γ-linolenate, 12(13)-EpOME, and 9,10-dihydroxy-12,13-epoxyoctadecanoate, with decreases ranging from 81.30 to 95.78% (Figure 10b). Meanwhile, three shared genes between the *α*-linolenic acid and linoleic acid pathways, including *LOX2S*, *CYP2J*, and *PLA2G*, were identified. BR and MT treatments significantly suppressed the expression of *LOX2S* and *PLA2G*, while *CYP2J* was also significantly downregulated, with transcript levels reduced by 82.89 to 91.64% compared with CK plants (Figure 10b). These results suggest that BR and MT may modulate cold-responses by coordinately regulating gene expression and metabolite accumulation in lipid-derived oxylipin pathways, thereby attenuating stress-associated lipid peroxidation and signaling activity.

### 2.10. Validation of RNA-Seq Data by Quantitative Real-Time PCR (qRT-PCR) Analysis

**Figure 11:**
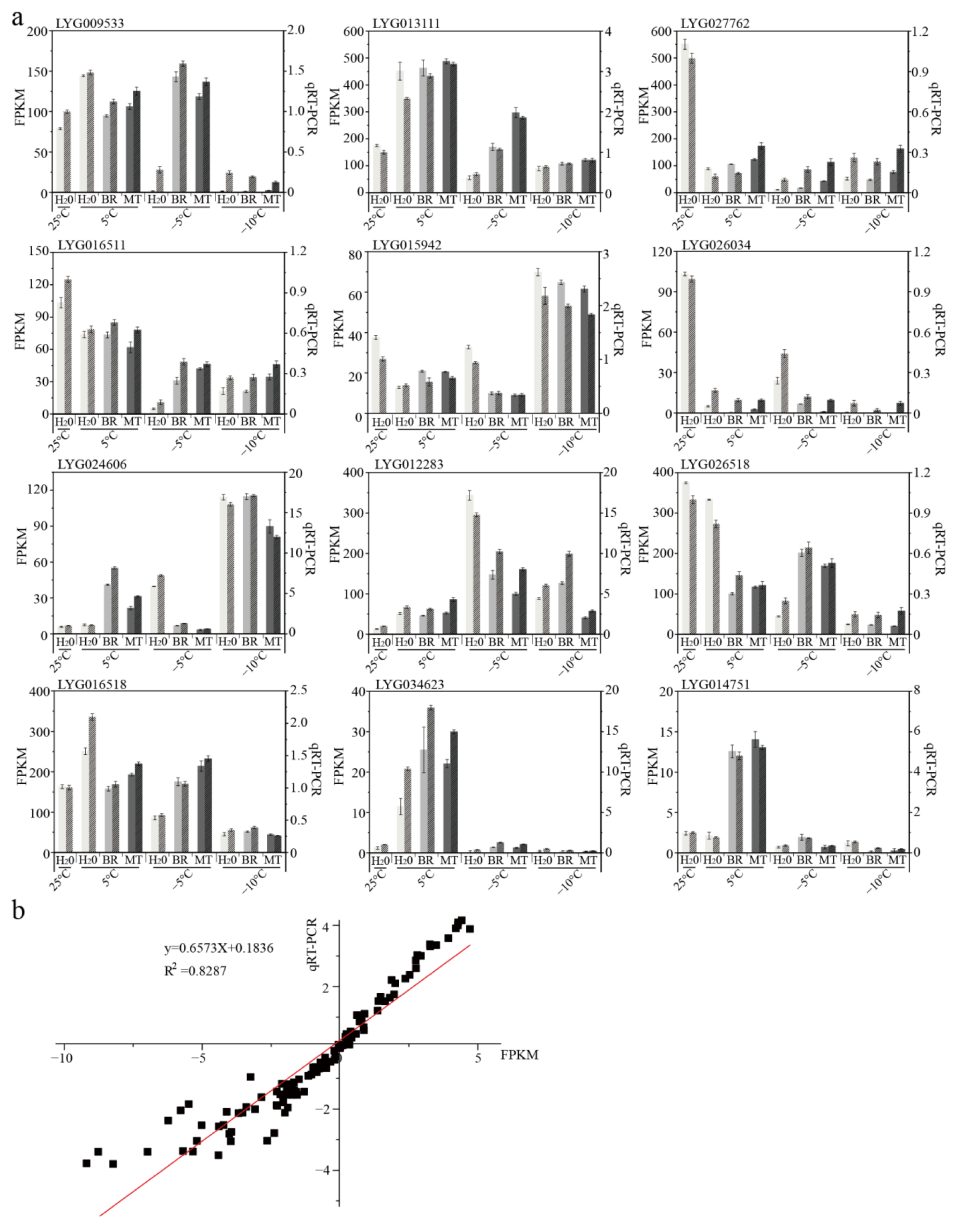
Expression of genes as determined by qRT-PCR. (**a**) Expression of genes as determined by qRT-PCR. Bar graphs represent mean ± standard deviation (SD). The gray bars represent FPKM values obtained from RNA-seq analysis, while the shaded bars represent relative gene expression levels determined by qRT-PCR. (**b**) Linear fitting of qRT-PCR data and FPKM data was performed after log2 normalizing their row data with CK data (25 °C, H_2_O-treated sample) as a reference, respectively. The *x*-axis represents FPKM data, and the *y*-axis represents qRT-PCR data.

To validate the reliability of the RNA-seq results, 12 representative DEGs were selected for qRT-PCR analysis, including four key TFs, four genes involved in phenylpropanoid and flavonoid biosynthesis, and four genes associated with *α*-linolenic acid and linoleic acid metabolism. The qRT-PCR results revealed that the expression trends of these genes were highly consistent with those obtained from RNA-seq analysis (Figure 11a). A strong positive correlation was observed between the two datasets, with a Pearson correlation coefficient of R^2^ = 0.83 (Figure 11b), indicating a high degree of concordance between RNA-seq and qRT-PCR results. These findings confirm the robustness and reliability of the transcriptome data, supporting its suitability for downstream analyses.

## 3. Discussion

### 3.1. Exogenous BR and MT Alleviate Low-Temperature Injury by Maintaining Photosynthesis and Enhancing Antioxidant Homeostasis

Low temperature stress is a major environmental constraint limiting plant growth, development, and ecological distribution. It primarily disrupts membrane integrity and photosynthetic stability, leading to excessive ROS accumulation and oxidative damage. Photosynthesis is highly sensitive to environmental fluctuations and depends on the coordinated regulation of chlorophyll content, photosynthetic performance, and chlorophyll fluorescence characteristics [36]. Low-temperature stress generally inhibits chlorophyll metabolism and accelerates pigment degradation, resulting in impaired chloroplast function and reduced photosynthetic efficiency. In the study, exposure to declining temperatures from −5 to −20 °C for 12 h caused progressive chlorosis and wilting in *O. fragrans* leaves, accompanied by a significant reduction in chlorophyll content (Figure 1 and Figure S5), indicating compromised photosynthetic capacity under cold stress. However, 50−200 μM BR and 0.5−2 μM MT treatments effectively alleviated chlorophyll degradation and maintained higher pigment levels under cold stress (Figure S5). Similar protective effects have been reported in cucumber, tomato (*Solanum lycopersicum* L.), and grape (*Vitis vinifera* ssp. *vinifera*) [26,27,31,37]. These findings suggest that BR and MT enhance cold tolerance by stabilizing chloroplast function, regulating pigment metabolism, and sustaining photosynthetic activity under adverse environmental conditions.

Excessive ROS accumulation is a hallmark of low-temperature-induced injury. Under cold stress, disruption of the photosynthetic electron transport chain promotes the overproduction of superoxide anion (O_2_·^−^), H_2_O_2_, and other ROS. Although ROS at moderate levels function as important signaling molecules that activate stress-responsive pathways, excessive ROS accumulation disrupts redox balance and causes oxidative damage to proteins, nucleic acids, and membrane lipids [38,39]. MDA, a major product of membrane lipid peroxidation, is widely used as a reliable indicator of oxidative membrane damage [40]. In this study, MDA, H_2_O_2_, and EL progressively increased following exposure to declining temperatures from −5 to −20 °C for 12 h, whereas the activities of antioxidant enzymes, including CAT, POD, and SOD, were significantly reduced (Figure 4a–f). Similar responses have been reported in maize (*Zea mays* L.) and barley (*Hordeum vulgare* L.) under cold stress [41,42,43]. These results indicate that low-temperature stress disrupts cellular redox homeostasis, impairs antioxidant defense capacity, and promotes membrane lipid peroxidation in *O. fragrans* leaves.

SOD, POD, and CAT constitute the core enzymatic antioxidant system responsible for maintaining cellular redox equilibrium. SOD converts O_2_·^−^ into H_2_O_2_, which is subsequently detoxified by CAT and POD into water and oxygen. The enhanced activities of these enzymes indicate improved ROS-scavenging efficiency and restoration of redox balance. In this study, exposure to declining temperatures from −5 to −20 °C for 12 h resulted in increased MDA, H_2_O_2_, and EL levels and reduced CAT, POD, and SOD activities, whereas exogenous BR (50–200 μM) and MT (0.5–2 μM) treatments decreased oxidative damage indicators and enhanced antioxidant enzyme activities (Figure 4), indicating that these regulators reinforce antioxidant capacity and alleviate cold-induced oxidative injury. Previous studies have shown that BR enhances antioxidant enzyme activities, induces stress-responsive gene expression, and promotes the accumulation of non-enzymatic antioxidants, such as ascorbate (AsA) and glutathione (GSH), thereby improving ROS detoxification efficiency [44,45]. Similarly, MT functions through both direct ROS scavenging and regulation of antioxidant-related signaling pathways [46]. Therefore, the protective effects of BR and MT may involve coordinated regulation of enzymatic antioxidants, redox signaling, and metabolic adjustment rather than a simple reduction in ROS accumulation.

Furthermore, WGCNA revealed that the darkorange2 module was positively correlated with CAT and SOD activities but negatively correlated with EL, MDA, and H_2_O_2_ levels (Figure 5a). This module was enriched in MAPK signaling, phenylpropanoid biosynthesis, and flavonoid biosynthesis pathways (Figure 5b), suggesting that the protective effects of 100 μM BR and 1 μM MT involve not only antioxidant enzyme regulation but also coordinated stress signaling and secondary metabolic reprogramming. These findings indicate that BR and MT alleviate low-temperature-induced physiological damage in *O. fragrans* by maintaining photosynthetic stability, enhancing antioxidant capacity, and coordinating stress-associated metabolic responses.

### 3.2. Reconstruction of Transcriptional Regulatory Networks During Low-Temperature Adaptation in O. fragrans

Plant adaptation to low-temperature stress is a complex biological process involving stress perception, transcriptional regulation, metabolic reprogramming, and physiological adjustment [34,47]. In the study, decreasing temperatures from 5 to −10 °C for 12 h triggered extensive transcriptional and metabolic reprogramming in *O. fragrans*. Notably, comparisons among different low-temperature treatments identified substantially more DEGs and DAMs than comparisons between BR/MT treatments and their corresponding CK at the same temperature (Tables S3 and S6), indicating that temperature variation is the predominant factor shaping transcriptomic and metabolic reprogramming. KEGG-based integrative analyses revealed that temperature-responsive DEGs and DAMs were mainly enriched in phenylpropanoid biosynthesis, flavonoid biosynthesis, starch and sucrose metabolism, flavone and flavonol biosynthesis, amino sugar and nucleotide sugar metabolism, linoleic acid metabolism, glycerolipid metabolism, and glycerophospholipid metabolism (Figure 7c,f,i). These pathways are associated with ROS scavenging, membrane lipid remodeling, energy metabolism regulation, and stress signaling transduction [48,49,50], suggesting that *O. fragrans* adapts to cold stress through coordinated regulation of transcriptional networks and metabolic pathways.

WGCNA further revealed distinct functional characteristics among cold-responsive gene modules. The lavenderblush3 module was enriched in carbon fixation in the Calvin cycle, photosynthesis–antenna proteins, pentose phosphate pathway, carotenoid biosynthesis, and nitrogen metabolism (Figure 5d), indicating a central role in maintaining photosynthetic efficiency, carbon assimilation, and energy homeostasis under low temperature [50,51]. Several TF families, including *WRKY*, *C2H2*, *bHLH*, *MYB*, and *DBB*, were identified within this module (Figure 5e). Previous studies have demonstrated that *WRKY* [52] and *MYB* [53,54] TFs participate in regulating stress-responsive and photosynthesis-associated genes, whereas *bHLH* [55,56] and *C2H2* [57] TFs contribute to light signaling and environmental adaptation. Therefore, the lavenderblush3 module may represent a regulatory network linking photosynthetic maintenance with stress adaptation. In contrast, the darkorange2 module was enriched in MAPK signaling, phenylpropanoid biosynthesis, flavonoid biosynthesis, and terpenoid metabolism (Figure 5b), suggesting its involvement in ROS homeostasis, stress signal transduction, and the activation of defensive secondary metabolism. This module contained key TFs belonging to the *ARF*, *EIL*, *B3*, and *bHLH* families (Figure 5c). *ARF* and *EIL* TFs are important components of auxin and ethylene signaling pathways, respectively, while *B3* and *bHLH* members participate in hormone regulation, secondary metabolism, and environmental responses [55,56,58,59,60]. These findings suggest that darkorange2 may integrate hormone-related signaling with antioxidant defense and secondary metabolic pathways, thereby contributing to enhanced cold tolerance in *O. fragrans*. However, because WGCNA primarily reflects gene coexpression patterns, further functional studies are required to determine whether these TFs directly regulate BR- or MT-mediated cold tolerance.

At the network level, ML-hGRN analysis identified multiple key TF families, including *ARF*, *EIL*, *bHLH*, *bZIP*, *GRAS*, and *MYB*, among which *LYG026518* (*ARF*) exhibited the highest network connectivity (Figure 6), suggesting that it may function as a central regulatory hub within the cold stress response network. Previous studies have shown that ARF proteins mediate crosstalk between auxin signaling and abiotic stress responses [58], whereas *EIL* are key components of ETH signaling pathways [59]. Both TF families are closely associated with stress tolerance regulation. In addition, *bHLH* [55,56], *bZIP* [61], and *GRAS* [62] TFs are widely involved in ROS scavenging, hormone signaling, and secondary metabolism. These TF families showed strong responsiveness to 100 μM BR and 1 μM MT treatments, suggesting that they may participate in the transcriptional reprogramming underlying exogenous regulator-mediated enhancement of cold adaptation.

### 3.3. Exogenous BR and MT Enhance Low-Temperature Tolerance by Regulating Phenylpropanoid, Flavonoid and Lipid Metabolism Pathways

Metabolic reprogramming represents a central adaptive strategy employed by plants under low-temperature stress, with activation of secondary metabolism and modulation of lipid metabolism playing essential roles in maintaining cellular homeostasis and enhancing stress tolerance [63,64]. The phenylpropanoid and flavonoid pathways generate diverse phenolic compounds with strong antioxidant properties [65], which contribute to ROS scavenging, cell wall reinforcement, membrane stabilization, and stress signal regulation. Therefore, activation of these pathways is widely recognized as a key mechanism underlying plant resistance to cold-induced oxidative damage. In the study, 100 μM BR and 1 μM MT treatments activated phenylpropanoid and flavonoid biosynthesis under −5 °C stress, accompanied by the upregulation of key structural genes, including *4CL*, *C4H*, *C3′H*, *COMT*, *CCoAOMT*, and *CSE* (Figure 9). This transcriptional activation was closely associated with the enhanced accumulation of chlorogenate, ferulate, sinapate, naringin, and 5-O-caffeoylshikimic acid (Figure 9). Among these metabolites, chlorogenate, ferulate, and sinapate are well-known phenolic antioxidants with strong ROS-scavenging capacity, effectively alleviating oxidative damage [65,66,67]. In addition, flavonoids such as naringin contribute to redox homeostasis and membrane protection. Phenylpropanoid-derived metabolites also serve in lignin biosynthesis and cell wall reinforcement, thereby enhancing tissue mechanical strength under cold stress [65,68]. These results indicate that BR- and MT-induced accumulation of phenolic acids and flavonoids provides a key metabolic basis for improved cold tolerance in *O. fragrans*.

Lipid metabolism reprogramming represents another essential component of cold adaptation. Low temperature decreases membrane fluidity, resulting in membrane rigidification and impaired cellular functions, whereas alterations in unsaturated fatty acids and their derivatives are closely associated with membrane remodeling and stress signaling. In this study, 100 μM BR and 1 μM MT treatments modulated *α*-linolenic acid and linoleic acid metabolism pathways under −5 °C stress (Figure 10), suggesting their involvement in maintaining lipid homeostasis during cold stress. In the *α*-linolenic acid metabolism pathway, BR and MT treatments markedly downregulated key genes, including *DOX*, *JMT*, *HPL*, *PLA2G*, and *PLA2G16*, accompanied by reduced accumulation of 12-OPDA and 9(S)-HPOT (Figure 10a). These metabolites are important intermediates of lipid oxidation and precursors of oxylipin signaling molecules [69,70]. Although oxylipins are commonly induced under abiotic stress to activate defense responses, their excessive accumulation is often associated with ROS overproduction and enhanced lipid peroxidation under severe stress conditions. Therefore, the reduced accumulation of 12-OPDA and 9(S)-HPOT in BR- and MT-treated plants may indicate alleviated oxidative stress and improved membrane homeostasis rather than a simple suppression of defense signaling. Similarly, in the linoleic acid metabolism pathway, BR and MT treatments suppressed *CYP2J* expression and significantly decreased the accumulation of multiple oxylipin-related metabolites, including 13-HODE, 13-HpODE, 9-OxoODE, γ-linolenate, 12(13)-EpOME, and 9,10-dihydroxy-12,13-epoxyoctadecanoate (Figure 10b). *CYP2J*-like genes belong to the cytochrome P450 superfamily and may participate in fatty acid oxidation and oxylipin biosynthesis [71], although their specific functions in *O. fragrans* remain unclear. Previous studies have demonstrated that P450-mediated fatty acid oxidation pathways are closely associated with stress responses, ROS metabolism, and membrane remodeling [72]. Collectively, the coordinated downregulation of *CYP2J*-like genes and reduced oxylipin accumulation suggest that BR and MT alleviate cold-induced lipid peroxidation by suppressing excessive oxylipin production and maintaining membrane stability. Overall, BR and MT enhance cold tolerance in *O. fragrans* through two complementary metabolic strategies: (i) activation of phenylpropanoid and flavonoid biosynthesis to enhance antioxidant capacity and ROS scavenging, and (ii) suppression of *α*-linolenic acid and linoleic acid metabolism to reduce oxylipin overproduction and lipid peroxidation. These coordinated metabolic adjustments collectively maintain cellular redox balance and membrane integrity, thereby improving plant adaptation to low-temperature stress.

### 3.4. Distinct Mechanisms Underlying BR- and MT-Mediated Enhancement of Cold Tolerance

Although BR and MT alleviated low-temperature-induced damage in *O. fragrans*, integrated transcriptomic and metabolomic analyses revealed differences in their regulatory scope and mechanistic emphasis. KEGG enrichment analysis further indicated that BR-responsive DEGs and DAMs were mainly associated with plant hormone signal transduction, glycerolipid metabolism, and ABC transporters (Figure 8f), whereas MT-responsive changes were additionally enriched in flavonoid biosynthesis, glycerophospholipid metabolism, and zeatin biosynthesis (Figure 8i). These results suggest that BR and MT may confer cold tolerance through partially distinct regulatory strategies. As a representative plant steroid hormone, BR primarily functions through modulation of hormone signaling networks. In this study, BR treatment significantly enriched the plant hormone signal transduction pathway, and key hormone-related TFs such as *ARF* and *EIL* were identified by WGCNA and ML-hGRN analyses (Figure 5 and Figure 6). These findings suggest that BR may enhance cold tolerance by activating phytohormone crosstalk networks (e.g., auxin and ETH signaling), thereby coordinating growth regulation and stress defense under low-temperature conditions. In contrast, MT, a multifunctional molecule with both signaling and antioxidant properties, appears to exert its effects mainly through redox homeostasis regulation and metabolic reprogramming. MT treatment markedly enhanced flavonoid biosynthesis and glycerophospholipid metabolism and induced a larger number of DEGs and DAMs. Together with the increased accumulation of phenolic and flavonoid metabolites and reduced ROS levels, these results suggest that MT not only directly scavenges ROS but also enhances antioxidant capacity and membrane lipid stability through metabolic reconfiguration, thereby improving cold adaptation.

Notably, despite these differences, BR and MT converged on common downstream processes, including maintenance of ROS homeostasis, suppression of lipid peroxidation, and activation of stress-responsive metabolism. The WGCNA-identified darkorange2 module and key TFs (*EIL*, *ARF*, *bHLH*, and *bZIP*) from the ML-hGRN network may serve as central regulatory hubs linking hormone signaling, redox regulation, and metabolic reprogramming. Overall, these findings indicate that BR- and MT-mediated cold tolerance is not independent but integrated within a shared cold-responsive regulatory network. BR primarily regulates hormone signaling to coordinate growth–defense balance, whereas MT predominantly enhances redox homeostasis and metabolic reprogramming. Together, they synergistically improve cold tolerance in *O. fragrans* by converging on common downstream protective mechanisms.

## 4. Materials and Methods

### 4.1. Plant Materials and Experimental Design

**Figure 12:**
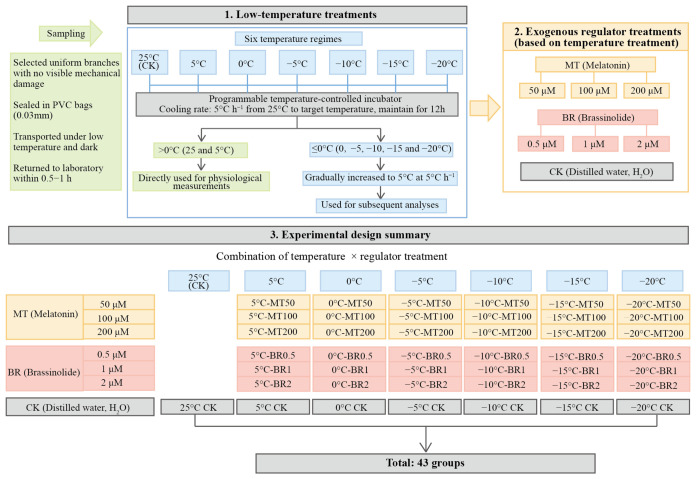
Experimental design for low-temperature stress and exogenous melatonin (MT) and brassinolide (BR) treatments.

*O. fragrans* plants approximately 20 years old, grown on the campus of Hubei University of Science and Technology (114°19′52″ E, 29°51′18″ N; Xianning, Hubei, China), were selected as experimental materials. In October 2025, one-year-old semi-lignified branches were collected from *O. fragrans* plants during the post-flowering vegetative growth stage. Branches exhibiting uniform growth and no visible mechanical damage were selected and immediately transported to the laboratory under low-temperature and dark conditions within 0.5–1 h for subsequent experiments. The collected branches were divided into 43 experimental groups (Figure 12). Samples maintained at 25 °C were used as the CK, while the remaining 42 groups were subjected to low-temperature and exogenous treatment experiments.

For low-temperature treatments, six temperature regimes (5, 0, −5, −10, −15, and −20 °C) were established. All samples were sealed in PVC preservation bags (0.03 mm thickness) and placed in a programmable temperature-controlled incubator (Haier, Qingdao, Shandong, China). Treatments were conducted for 12 h, with a cooling rate of 5 °C h^−1^ from 5 °C to the target temperature. After treatment, samples exposed to temperatures above 0 °C (25 and 5 °C) were directly used for physiological measurements. For samples treated at ≤0 °C, temperature was gradually increased back to 5 °C at a rate of 5 °C h^−1^ prior to further analysis. Based on each temperature treatment, exogenous regulator treatments were conducted using MT (50, 100, and 200 μM) [19,25] or BR (0.5, 1, and 2 μM) [73], and distilled water (H_2_O) as the CK. Each treatment included three biological replicates, with six branches per replicate. Exogenous solutions were uniformly sprayed onto leaf surfaces prior to low-temperature exposure. After treatment, leaf samples were harvested, immediately frozen in liquid nitrogen, and stored at −80 °C for subsequent analyses.

### 4.2. EL, MDA, H_2_O_2_ Determination and Histochemical Staining

O_2_·^−^ was visualized using NBT staining (Coolaber, Beijing, China). Fresh leaves were immersed in 0.1% NBT solution (10 mM phosphate buffer, pH 7.8) and incubated at 37 °C in the dark for 1–2 h. During this process, O_2_·^−^ reacts with NBT to form a blue formazan precipitate. After incubation, samples were rinsed with distilled water to remove excess dye and decolorized in 95% ethanol until the background became clear, allowing visualization of formazan deposition. H_2_O_2_ accumulation was detected using DAB staining (Coolaber). Leaf samples were immersed in 1 mg mL^−1^ DAB solution (pH 3.8) and incubated at 37 °C in the dark for 8–12 h. H_2_O_2_ reacts with DAB to form a brown precipitate. Samples were subsequently decolorized in 95% ethanol, and images were recorded for analysis.

H_2_O_2_ content was determined using the titanium sulfate method [74]. EL was determined according to Tsarouhas et al. [75] with minor modifications. MDA content was determined using the thiobarbituric acid (TBA) method [76].

### 4.3. Determination of Chlorophyll Content

Chlorophyll content was determined using the 95% ethanol extraction method. Chlorophyll concentrations were calculated according to the equations of Lichtenthaler and Wellburn [77].

### 4.4. Antioxidant Enzyme Activity Assays

Approximately 0.2 g of fresh plant tissue was homogenized in 2 mL of 50 mM phosphate buffer (pH 7.0) under ice-cold conditions. The homogenate was centrifuged at 10,000× *g* for 15 min at 4 °C, and the resulting supernatant was collected as the crude enzyme extract. POD activity was determined using the guaiacol method [78]. CAT activity was measured spectrophotometrically based on the decomposition rate of H_2_O_2_ [79]. SOD activity was determined using the NBT photoreduction method [80].

### 4.5. Transcriptome Analysis

Total RNA was extracted using the HiPure Plant RNA Mini Kit (Magen Bio., Guangzhou, Guangdong, China) according to the manufacturer’s instructions. Library construction and Illumina NovaSeq sequencing were performed by Igenebook Technology Co., Ltd. (Wuhan, Hubei, China). Raw sequencing data were subjected to QC using FastQC v0.11.5 [81], including removal of adapter sequences, low-quality reads, and reads containing ambiguous nucleotides (N bases), yielding high-quality clean reads. Clean reads were aligned to the *O. fragrans* “Liuye Jingui” reference genome [82] using HISAT2 v2.0.1-beta [83]. Gene-level read counts were obtained using FeatureCounts v1.6.0 [84] based on the genome annotation file. Gene expression levels were normalized and calculated as fragments per kilobase of transcript per million mapped reads (FPKM) to enable comparisons among samples.

Pearson correlation coefficients were calculated based on gene expression profiles to assess sample relationships, and correlation heatmaps were generated accordingly. DEGs were identified using the edgeR package (http://bioconductor.org, accessed on 4 June 2026) [85] in R. Genes with a false discovery rate (FDR) < 0.05 and |log_2_ fold change| > 1 were considered significantly differentially expressed. KEGG pathway enrichment analysis [86] was performed, and genes mapped to KEGG pathways were tested for enrichment using a significance threshold of *p* < 0.05.

### 4.6. Weighted Gene Coexpression Network Analysis

WGCNA was performed using the WGCNA R package v1.1.25. Genes with a SD < 0.5 across samples were filtered prior to network construction. A signed network was constructed using an adaptive soft-thresholding power to ensure scale-free topology. The minimum module size was set to 20 genes, and the module merging threshold was set to 0.25. The cut height for dynamic tree cutting was set to 2. Pearson correlation coefficients were calculated to evaluate relationships between modules, module eigengenes, and physiological traits. Hub genes were identified based on intramodular connectivity, and the top 100 hub genes were selected for network visualization.

### 4.7. Multi-Layer Gene Regulatory Network Analysis

A backward elimination-based random forest approach (BWERF) was employed to construct an ML-hGRN [87]. In this framework, genes associated with target pathways were first defined as the base layer (Level 1). A random forest model was then applied to identify TFs showing significant regulatory relationships with these target genes, which were subsequently assigned to Level 2. Next, TFs identified in Level 2 were removed from the candidate regulator pool and redefined as the new base layer, while the remaining genes were used as candidate regulators to iteratively construct higher network layers. This procedure was repeated until a predefined number of layers was reached or no additional TFs with significant regulatory associations could be identified.

In this study, 120 DEGs shared among the 5, −5, and −10 °C treatments were used as structural genes, while 895 TFs identified from the same datasets were used as candidate regulatory factors. The ML-hGRN was constructed based on these datasets. For each random forest model, the number of trees was set to 1000 (ntree = 1000), and variance reduction was used as the splitting criterion for node construction. During iterative modeling, TFs were ranked in descending order based on their importance scores, and the least informative TFs were removed using an elimination ratio of 1/10 per iteration. The remaining TFs were then used to retrain the model. This iterative process continued until only a single TF remained, resulting in a three-layer hierarchical regulatory network.

To reduce stochastic variation and potential overfitting, the model was independently run 30 times for each dataset. The importance score of each TF–target pair was averaged across the 30 runs, and regulatory interactions were ranked accordingly. Only TF–target interactions consistently exhibiting high importance scores across repeated runs were retained as putative regulatory edges and used to construct the final multi-layer gene regulatory network.

### 4.8. Metabolome Analysis

Metabolite extraction and detection were performed according to previously described methods with minor modifications [88]. Briefly, freeze-dried samples were ground into powder, and metabolites were extracted using precooled 70% methanol containing internal standards. After centrifugation and filtration, the extracts were subjected to ultra-performance liquid chromatography coupled with tandem mass spectrometry (UPLC–MS/MS) analysis. Chromatographic separation was performed using a Waters ACQUITY UPLC HSS T3 column (Waters Co., Milford, MA, USA). The mobile phase consisted of solvent A (ultrapure water with 0.1% formic acid) and solvent B (acetonitrile with 0.1% formic acid). The column temperature was maintained at 40 °C, with a flow rate of 0.40 mL min^−1^ and an injection volume of 4 μL. The total run time was 10 min. The gradient elution program was as follows: 0 min, 95% A; 5 min, 35% A; 6 min, 1% A; and 7.6 min, returned to 95% A. Mass spectrometry data were acquired under both positive and negative electrospray ionization modes. The spray voltages were set to 5000 V (positive mode) and 4000 V (negative mode), respectively. The ion source temperature was maintained at 550 °C, with curtain gas at 35 psi, ion source gas1 at 50 psi, and ion source gas2 at 60 psi. The declustering potential was set to 60 V. Collision energies were set at 10 V for MS1 and 30 V for MS/MS, with a collision energy spread of 15 V. The mass scan ranges were *m*/*z* 50–1250 for MS1 and *m*/*z* 25–1250 for MS/MS.

Raw mass spectrometry data were processed using the XCMS package v3.23 for peak detection, alignment, and retention time correction. Missing values were filtered and imputed, and peak areas were normalized using a support vector regression (SVR) method. Metabolite identification was performed by matching against in-house and public databases, predicted libraries, and the metDNA platform. Metabolites with a combined identification score > 0.5 and a coefficient of variation (CV) < 0.5 in QC samples were retained. QC samples were prepared by pooling equal aliquots of all extracts and were injected every 10 samples to monitor system stability. Total ion chromatograms (TICs) of QC samples were overlaid to assess instrument repeatability, and Pearson correlation analysis was performed among QC samples.

Multivariate statistical analyses, including PCA, partial least squares discriminant analysis (PLS-DA), and orthogonal partial least squares discriminant analysis (OPLS-DA), were conducted on all samples, including QC samples. Metabolites with variable importance in projection (VIP) > 1 and |log_2_ fold change| ≥ 1 were considered DAMs. Functional annotation of metabolites was performed using the KEGG database [86], followed by enrichment analysis of DAMs.

### 4.9. Integrated Transcriptome and Metabolome Analysis

DEGs and DAMs from pairwise comparisons were jointly subjected to pathway enrichment analysis. Coenrichment bubble plots were generated using the Metware Cloud platform (https://cloud.metware.cn), and the top 25 pathways ranked by Q-value were selected for visualization. In addition, pathway maps of key metabolic pathways were constructed to illustrate integrated regulatory relationships between genes and metabolites.

### 4.10. Quantitative Real-Time PCR Analysis

Total RNA was extracted from samples and reverse-transcribed into first-strand cDNA. qRT-PCR was performed using ChamQ^TM^ SYBR^®^ qPCR Master Mix Kit (Vazyme, Nanjing, Jiangsu, China) on a TianLong Gentier 96E real-time PCR system (Tianlong Technology, Xi’an, Shanxi, China). Each reaction included three biological replicates and three technical replicates. Primers were designed using Primer Premier 5.0 software (Premier Biosoft, San Francisco, CA, USA) (Table S7). *O. fragrans* actin (*OfActin*) and *OfRAN1* were used as internal reference genes [89]. Relative gene expression levels were calculated using the 2^−ΔΔCt^ method [90].

### 4.11. Statistical Analysis

All experimental data were analyzed using SPSS software version 26.0 (IBM Co., Armonk, NY, USA). One-way analysis of variance (ANOVA) followed by Duncan’s multiple range test was performed to determine statistically significant differences among different temperature treatments within the same treatment group at *p* < 0.05. Student’s *t*-test was conducted to evaluate significant differences between each treatment and the corresponding CK under the same temperature condition at *p* < 0.05. Data are presented as mean ± SD, with three independent biological replicates for each experiment. Graphical representations were generated using Origin 2018 software (OriginLab Co., Northampton, MA, USA).

## 5. Conclusions

This study integrates physiological, transcriptomic, and metabolomic analyses to systematically elucidate the mechanisms by which BR and MT enhance cold tolerance in *O. fragrans*. BR and MT alleviated low-temperature-induced oxidative damage by reducing EL, MDA, and H_2_O_2_ accumulation and enhancing antioxidant enzyme activities. WGCNA and ML-hGRN analyses identified key TFs, including *ARF*, *EIL*, *bHLH*, and *GRAS*, indicating that hormone signaling and stress-responsive regulatory networks contribute to cold tolerance. Integrated transcriptomic and metabolomic analyses further revealed that BR and MT promote phenylpropanoid and flavonoid biosynthesis and regulate *α*-linolenic acid and linoleic acid metabolism, thereby enhancing secondary metabolism and maintaining membrane lipid homeostasis. Notably, BR primarily improved cold tolerance through hormone-related regulatory networks, whereas MT mainly acts via redox regulation and metabolic reprogramming. Overall, this study provides new insights into hormone-mediated cold adaptation in woody ornamental plants and offers a theoretical basis for improving cold-resilient cultivation and breeding strategies in *O. fragrans*. Future studies focusing on functional validation of key TFs, lipidomic analyses, and time-series multi-omics approaches will further clarify the dynamic regulatory mechanisms underlying cold stress responses.

## Data Availability

The transcriptome sequencing data generated in this study have been deposited in the NCBI Sequence Read Archive (SRA) under BioProject accession number PRJNA1492739.

## Supplementary Materials

The following supporting information can be downloaded at: https://www.mdpi.com/article/10.3390/plants15152404/s1. Figure S1: sample quality assessment; Figure S2: metabolite class composition donut plots; Figure S3: quality control (QC) assessment of samples; Figure S4: PCA score plot of mass spectrometry data from all sample groups and QC samples; Figure S5: chlorophyll content. Table S1: statistical summary of the cleaned dataset; Table S2: summary of alignment results; Table S3: summary of differentially expressed genes (DEGs) across all samples; Table S4: expression analysis of transcription factors (TFs) at different levels; Table S5: summary of DEGs across all samples; Table S6: statistics of differentially accumulated metabolites (DAMs) across all samples; Table S7: primers used in this study.
