# Supplementary material for: Melatonin and Brassinolide Enhance Cold Tolerance in Osmanthus fragrans: Insights from Integrated Physiological and Multi-Omics Analyses
Source: Plants (Basel). 2026 Aug 6;15(15):2404. doi: 10.3390/plants15152404 (PMC13468045; doi:10.3390/plants15152404)
Supplement: Supplementary file 1 [file plants-15-02404-s001.zip › plants-4462297-supplementary.pdf]

Table S1. Statistical summary of the cleaned dataset.

Sample	Raw reads	Raw bases	Clean reads	Clean bases	Clean ratio (%)	Q20 (%)	Q30 (%)	GC (%)
CK_1	44,645,266	6,696,789,900	44,622,620	6,677,123,504	99.95	98.84	97.10	41.45
CK_2	41,259,112	6,188,866,800	41,238,452	6,161,469,413	99.95	98.85	97.08	42.15
CK_3	51,749,922	7,762,488,300	51,722,830	7,731,650,480	99.95	98.82	97.04	41.32
5BR_1	39,150,606	5,872,590,900	39,130,912	5,852,135,885	99.95	98.75	96.88	40.96
5BR_2	49,519,248	7,427,887,200	49,491,056	7,404,525,815	99.94	99.05	97.29	42.09
5BR_3	42,261,824	6,339,273,600	42,240,660	6,307,628,705	99.95	98.74	96.85	41.36
5H2O_1	40,022,652	6,003,397,800	39,998,864	5,982,000,667	99.94	98.88	97.16	42.21
5H2O_2	46,867,336	7,030,100,400	46,841,048	7,000,363,098	99.94	98.81	97.02	41.29
5H2O_3	43,505,942	6,525,891,300	43,481,730	6,502,172,457	99.94	98.75	96.84	41.32
5MT_1	38,218,346	5,732,751,900	38,200,306	5,711,235,739	99.95	98.84	97.05	40.85
5MT_2	44,172,044	6,625,806,600	44,149,144	6,599,136,470	99.95	98.78	96.95	40.77
5MT_3	39,669,932	5,950,489,800	39,648,732	5,926,144,054	99.95	98.70	96.74	40.90
−10BR_1	42,232,492	6,334,873,800	42,208,452	6,312,198,286	99.94	98.85	96.97	43.73
−10BR_2	38,442,088	5,766,313,200	38,420,456	5,747,570,292	99.94	98.97	97.15	44.56
−10BR_3	38,050,242	5,707,536,300	38,029,292	5,687,604,232	99.94	98.74	96.71	43.75
−10H2O_1	43,850,902	6,577,635,300	43,830,376	6,555,242,565	99.95	98.89	97.18	42.70
−10H2O_2	41,119,638	6,167,945,700	41,100,246	6,147,153,770	99.95	98.80	97.00	40.85
−10H2O_3	41,989,726	6,298,458,900	41,970,268	6,277,606,242	99.95	99.01	97.49	42.96
−10MT_1	36,471,994	5,470,799,100	36,452,866	5,445,834,612	99.95	98.80	96.99	43.52
−10MT_2	41,015,180	6,152,277,000	40,994,176	6,127,713,718	99.95	98.70	96.76	41.72
−10MT_3	37,716,472	5,657,470,800	37,698,766	5,639,681,993	99.95	98.85	97.07	43.32
−5BR_1	41,632,482	6,244,872,300	41,612,388	6,222,344,368	99.95	98.77	96.89	41.68
−5BR_2	40,670,788	6,100,618,200	40,650,562	6,078,817,072	99.95	98.68	96.70	39.95
−5BR_3	38,946,654	5,841,998,100	38,926,186	5,813,523,351	99.95	98.69	96.71	42.08
−5H2O_1	38,331,548	5,749,732,200	38,312,200	5,729,085,853	99.95	98.78	96.94	41.60
−5H2O_2	37,253,206	5,587,980,900	37,233,384	5,555,596,805	99.95	98.76	96.89	42.50
−5H2O_3	41,335,478	6,200,321,700	41,315,560	6,179,006,896	99.95	98.78	96.94	41.53
−5MT_1	37,946,490	5,691,973,500	37,927,720	5,669,524,447	99.95	98.76	96.86	42.01
−5MT_2	49,086,640	7,362,996,000	49,058,558	7,335,655,784	99.94	99.04	97.27	41.91
−5MT_3	46,819,620	7,022,943,000	46,795,242	6,997,233,940	99.95	98.69	96.73	40.99

Table S2. Summary of alignment results.

Sample	Total reads	Mapped reads	Map rate (%)	Uniq	Paired	Single	selfAND mate	mapDif fCHR
CK_1	44,622,620	40,969,704	91.81	39,548,467	37,443,968	1,977,222	38,992,482	218,882
CK_2	41,238,452	37,926,591	91.97	36,618,819	34,678,652	1,790,967	36,135,624	232,552
CK_3	51,722,830	47,468,162	91.77	45,785,751	43,432,254	2,215,822	45,252,340	241,166
5BR_1	39,130,912	35,230,394	90.03	34,076,039	32,084,564	1,909,198	33,321,196	188,838
5BR_2	49,491,056	45,160,073	91.25	43,671,505	41,447,966	2,119,617	43,040,456	225,640
5BR_3	42,240,660	38,078,783	90.15	36,839,966	34,688,072	2,038,953	36,039,830	226,094
5H_2O_1	39,998,864	36,483,990	91.21	35,040,721	33,192,556	1,757,102	34,726,888	195,284
5H_2O_2	46,841,048	42,445,045	90.62	40,770,389	38,464,448	2,202,005	40,243,040	226,940
5H_2O_3	43,481,730	39,457,339	90.74	37,894,662	35,712,326	2,084,683	37,372,656	220,046
5MT_1	38,200,306	34,622,057	90.63	33,505,372	31,578,608	1,831,297	32,790,760	193,748
5MT_2	44,149,144	39,935,172	90.46	38,645,971	36,487,514	2,069,622	37,865,550	196,532
5MT_3	39,648,732	35,904,228	90.56	34,765,173	32,771,794	1,907,170	33,997,058	186,950
−10BR_1	42,208,452	32,214,960	76.32	31,330,330	29,765,622	1,495,360	30,719,600	123,484
−10BR_2	38,420,456	28,843,949	75.07	28,055,031	26,715,162	1,272,519	27,571,430	114,026
−10BR_3	38,029,292	28,571,078	75.13	27,807,007	26,321,366	1,418,762	27,152,316	118,100
−10H_2O_1	43,830,376	35,658,360	81.36	34,749,252	32,894,054	1,776,266	33,882,094	142,222
−10H_2O_2	41,100,246	34,166,234	83.13	33,318,902	31,387,310	1,867,096	32,299,138	128,834
−10H_2O_3	41,970,268	33,915,795	80.81	33,060,643	31,342,152	1,648,103	32,267,692	126,206
−10MT_1	36,452,866	29,774,434	81.68	28,930,105	27,484,772	1,385,428	28,389,006	110,252
−10MT_2	40,994,176	34,165,620	83.34	33,232,031	31,422,588	1,750,926	32,414,694	123,740
−10MT_3	37,698,766	30,875,053	81.90	30,006,194	28,476,054	1,464,431	29,410,622	122,522
−5BR_1	41,612,388	37,796,350	90.83	36,482,654	34,444,174	1,927,600	35,868,750	212,372
−5BR_2	40,650,562	36,550,038	89.91	35,319,332	33,085,844	2,143,402	34,406,636	199,324
−5BR_3	38,926,186	35,329,865	90.76	34,131,371	32,256,206	1,784,877	33,544,988	181,760
−5H_2O_1	38,312,200	34,211,581	89.30	33,212,982	31,264,388	1,850,049	32,361,532	170,996
−5H_2O_2	37,233,384	33,356,725	89.59	32,330,499	30,552,000	1,674,231	31,682,494	168,820
−5H_2O_3	41,315,560	37,076,310	89.74	35,971,661	33,944,872	1,929,878	35,146,432	172,298
−5MT_1	37,927,720	34,451,162	90.83	33,326,167	31,479,592	1,757,302	32,693,860	181,312
−5MT_2	49,058,558	44,718,061	91.15	43,263,550	40,934,612	2,200,573	42,517,488	243,684
−5MT_3	46,795,242	42,232,516	90.25	40,879,013	38,394,624	2,363,878	39,868,638	243,334

Table S3. Summary of differentially expressed genes (DEGs) across all samples.

Sample ID	Total	UP num	DOWN num
5BR_vs_5H_2O	7630	3677	3953
5MT_vs_5H_2O	4245	1934	2311
−5BR_vs_−5H_2O	7754	3615	4139
−5MT_vs_−5H_2O	10,462	4876	5586
−10BR_vs_−10H_2O	2183	686	1497
−10MT_vs_−10H_2O	2760	1054	1706
−10H_2O_vs_5H_2O	13,108	6801	6307
−5H_2O_vs_5H_2O	10,491	5488	5003
−10H_2O_vs_−5H_2O	4780	1578	3202
−5BR_vs_5BR	6882	3419	3463
−10BR_vs_5BR	9108	4417	4691
−10BR_vs_−5BR	11,450	5709	5741
−5MT_vs_5MT	6602	3069	3533
−10MT_vs_5MT	10,773	5562	5211
−10MT_vs_−5MT	13,762	7269	6493
All	20,149		

Table S4. Expression analysis of transcription factors (TFs) at different levels.

	CK	5H_2O	5BR	5MT	−5H_2O	−5BR	−5MT	−10H_2O	−10BR	−10MT
**Level 1**										
LYG029284	0.95	10.74	1.97	6.74	513.60	4.69	7.71	754.29	761.70	302.88
LYG003351	78.17	699.32	325.46	584.62	2379.45	944.24	611.70	1327.79	1531.86	553.41
LYG004233	61.50	88.11	57.25	136.13	276.22	30.84	27.88	541.97	444.46	186.96
LYG004610	8.62	8.18	5.96	6.42	53.64	14.28	13.93	39.01	45.65	32.31
LYG036723	10.06	29.25	9.72	20.19	158.63	30.33	52.42	155.20	178.29	38.78
LYG038417	5.16	35.26	22.40	40.60	86.51	15.31	20.09	92.09	121.67	66.96
LYG007210	1.79	73.73	24.52	73.27	1035.23	318.31	344.05	1412.43	1412.14	320.31
LYG007339	20.87	138.25	159.60	156.77	940.67	244.87	248.18	623.81	754.95	239.56
LYG008618	0.00	0.74	0.21	0.07	6.94	1.83	3.79	29.80	13.31	1.91
LYG012465	4.38	181.52	127.10	303.56	1181.20	276.85	183.60	652.95	651.50	228.71
**Level 2**										
LYG001966	0.05	0.21	0.04	0.19	19.06	0.76	0.58	9.24	4.43	1.41
LYG018505	10.49	13.93	1.64	10.85	33.48	4.26	7.86	21.63	22.28	7.76
LYG019160	124.00	413.24	231.35	381.36	2414.03	241.46	221.66	2089.10	2017.70	790.90
LYG019979	201.47	457.25	493.37	459.58	2063.52	140.37	116.37	1282.93	1216.03	584.88
LYG020979	42.39	313.72	68.22	228.41	961.66	123.44	107.80	1271.97	1122.54	459.99
LYG021763	0.00	3.97	0.40	0.90	345.27	37.92	8.27	18.72	8.59	3.53
LYG023120	2.39	4.03	1.23	1.62	19.02	0.31	0.14	5.38	10.23	1.83
LYG023617	75.49	503.17	306.24	670.19	1882.64	499.51	384.32	1711.18	1741.96	922.18
LYG027126	8.42	159.24	21.06	67.01	1089.90	361.05	223.00	508.35	96.75	103.67
LYG028176	0.22	16.23	2.43	1.51	1317.00	195.35	112.81	318.53	321.07	67.97
LYG029919	44.41	222.41	68.49	109.22	784.26	143.37	143.33	572.41	525.95	279.70
LYG030379	14.27	22.82	23.52	21.09	8.02	28.17	37.62	8.48	9.86	11.29

LYG033234	43.04	204.44	137.49	190.49	654.82	125.76	111.76	372.77	338.79	133.56
LYG004259	8.43	11.65	16.12	9.83	90.58	30.56	28.15	71.20	77.69	40.60
LYG033714	2.55	1.59	1.46	1.56	7.84	2.68	1.90	4.08	3.42	2.12
LYG037698	98.43	56.60	49.44	38.25	10.72	26.59	25.41	17.05	17.32	22.98
LYG005346	6.20	14.37	16.24	15.82	53.67	3.39	2.39	19.67	18.44	15.44
LYG007386	7.29	18.27	7.76	17.98	108.43	24.04	11.01	80.61	40.64	15.13
LYG014708	9.25	6.92	11.49	13.83	1.83	4.93	5.61	1.30	0.64	2.48
LYG016405	2.04	12.49	12.15	9.53	92.94	19.25	15.13	79.09	49.03	14.31
**Level 3**										
LYG000491	297.95	2611.44	995.83	1578.47	6243.67	576.50	558.71	4038.68	3218.01	1110.13
LYG001665	15.39	11.18	45.72	24.22	9.16	22.83	18.42	6.59	9.59	13.81
LYG002617	0.74	85.42	6.52	31.54	60.63	34.72	24.41	94.09	28.17	4.78
LYG018604	40.13	88.69	131.61	101.39	78.16	134.89	139.29	111.76	181.58	159.95
LYG021065	46.08	100.09	91.23	92.45	36.55	98.92	109.85	38.60	42.23	43.97
LYG023018	124.44	4.57	45.59	38.94	2.45	13.30	2.60	6.38	0.42	0.43
LYG023436	28.38	32.29	23.18	24.52	13.15	30.11	41.69	16.43	20.97	22.93
LYG024697	46.09	16.28	48.20	33.11	44.84	28.12	21.56	88.62	96.05	107.30
LYG025718	163.88	102.92	101.72	87.81	84.48	148.72	175.31	80.24	114.39	99.66
LYG026518	374.92	333.23	100.22	117.05	44.27	201.59	169.49	24.52	23.26	20.15
LYG027643	7.55	37.10	30.21	49.39	123.03	46.62	83.89	106.44	30.40	14.57
LYG030178	343.20	451.74	40.49	128.30	252.47	221.43	151.39	7.74	3.54	2.18
LYG031694	54.87	61.34	45.46	36.92	33.74	153.89	258.68	11.99	15.91	17.95
LYG004261	73.42	97.25	42.72	56.63	19.09	86.39	102.19	18.81	26.52	29.02
LYG034789	32.05	10.60	41.23	38.32	13.15	20.61	16.39	2.91	0.72	1.58
LYG035545	13.20	23.67	9.65	14.39	4.29	6.89	6.03	5.13	2.86	3.40
LYG036671	9.99	40.45	18.03	34.06	116.56	34.35	25.24	68.08	60.15	39.75
LYG037394	0.10	3.93	0.64	0.82	736.47	55.76	26.37	342.20	145.06	22.04
LYG037710	55.92	28.72	59.00	53.21	182.90	38.51	41.44	347.78	297.68	122.37
LYG038346	393.68	366.29	170.50	242.27	129.76	210.70	191.06	90.26	78.25	69.44
LYG038385	83.64	19.23	18.04	16.39	2.98	31.23	19.51	8.81	9.16	11.46
LYG005865	6.14	2.77	17.32	12.54	1.82	4.57	2.68	2.26	0.47	1.19
LYG006184	9.76	58.32	24.21	49.86	195.77	21.32	14.44	292.17	162.82	77.80
LYG008109	5.87	16.78	2.84	8.92	148.24	21.05	13.69	97.38	68.19	11.51
LYG008617	1.29	18.80	3.51	5.82	870.87	73.60	55.62	988.15	431.05	69.33
LYG010377	53.45	53.01	41.44	43.27	19.96	46.95	56.36	22.06	25.75	27.85
LYG011749	25.40	87.78	23.69	30.08	208.98	37.46	47.46	237.82	86.48	58.82
LYG011853	3.52	31.84	2.77	8.53	354.13	19.94	9.97	273.16	170.53	22.22
LYG011909	175.08	411.83	132.04	252.66	292.48	126.26	202.80	34.58	50.33	17.98
LYG012134	323.91	291.56	202.44	287.77	1179.57	493.16	455.79	758.14	554.52	233.27
LYG013104	18.61	17.11	28.93	22.01	104.15	24.79	28.82	99.84	82.62	48.40
LYG013932	38.59	40.55	36.79	48.31	149.04	40.82	31.69	89.10	79.34	63.58
LYG014735	7.29	10.50	27.51	15.57	4.00	7.18	11.58	5.34	6.85	9.83
LYG015018	8.24	33.01	16.84	30.10	151.93	49.65	51.67	106.25	94.18	81.18
LYG016184	2473.61	1265.25	337.53	778.68	1229.21	47.49	24.54	1356.26	854.37	442.15
LYG016638	11.71	0.85	3.36	2.69	0.63	1.39	1.16	0.73	0.36	1.63
LYG016978	35.69	66.74	15.75	43.32	82.00	22.23	15.32	27.52	33.39	9.75
LYG017155	28.24	18.25	12.48	14.21	7.11	15.23	16.58	8.06	11.42	14.62
LYG017360	60.89	652.00	97.04	174.88	109.71	230.02	150.22	5.85	35.35	20.46
LYG017378	1.53	14.06	0.49	1.47	18.13	4.31	1.86	5.75	8.51	8.61

Table S5. Summary of DEGs across all samples.

Ion mode	All	Positive	Negative
Number of metabolites	2936	1482	1454

Table S6. Statistics of differentially accumulated metabolites (DAMs) across all samples.

Group name	All sig diff	Down regulated	Up regulated
5BR_vs_5H_2O	184	90	94
5MT_vs_5H_2O	205	68	137
−5BR_vs_−5H_2O	721	278	443
−5MT_vs_−5H_2O	718	280	438
−10BR_vs_−10H_2O	486	312	174
−10MT_vs_−10H_2O	567	306	261
−5H_2O_vs_5H_2O	729	480	249
−10H_2O_vs_5H_2O	779	455	324
−10H_2O_vs_−5H_2O	481	209	272
−5BR_vs_5BR	203	114	89
−10BR_vs_5BR	772	504	268
−10BR_vs_−5BR	712	460	252
−5MT_vs_5MT	342	260	82
−10MT_vs_5MT	848	569	279
−10MT_vs_−5MT	685	398	287

Table S7. Primers used in this study.

Gene	Forward primer	Reverse primer
LYG009533	AAGATGCCGTTAGGAGGAAGTT	CCACATGCCCCAGATTTTATT
LYG013111	TCAAATCTGCCATTGAACTCG	ATTCTTCGTCGGAATCTGCTC
LYG027762	AACCTCCACGATGCCAAACT	AATTCCTCGGGATTCTTCCA
LYG016511	CTCCGATTTCGTCTGCCTCC	TGCCTATCCGCCAACTGCT
LYG015942	TCGGTAATGTGCTCTGTCGTTG	GGCATCTCGGCGGTTCA
LYG026034	CATTTGAAGTGAAGGCAGAGGT	TTCGCTACATTGTATCCATCGTC
LYG024606	GAGGCGAGGGAAGTTCTCAG	CCAGTAGTGGAATTGGGGTGT
LYG012283	GTGCTCGTCGGAGATTTCATG	TTGGCAACCTCAGCGTCA
LYG026518	TATTTCCCGCAGGGTCGC	GAGTTGGTGTCTGCTGGCTCATA
LYG016518	AGATCCGCAAACTTGTCCAGC	TGAATACGACCCACTCCCACC
LYG034623	TCTTTTGCCCCAACTTGCC	ATTGAGACAGAGCTGGAGGATGA
LYG014751	GTCGAGCATCGTTTGGTCATG	GCCATCTTCCTCCGTAGCCT
LYG009533	AAGATGCCGTTAGGAGGAAGTT	CCACATGCCCCAGATTTTATT

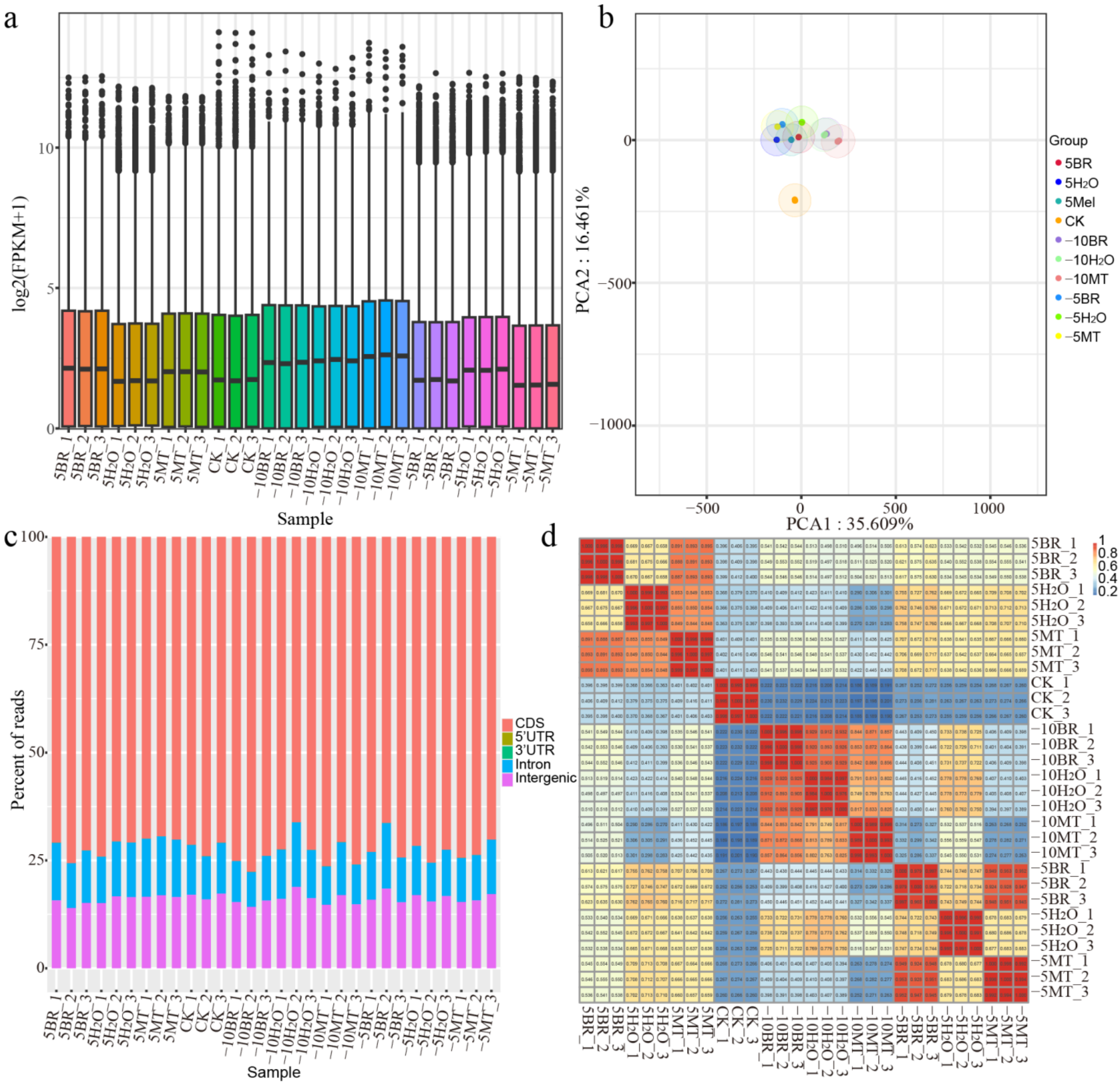

Figure S1. Sample quality assessment. (a) FPKM distribution of all samples. Gene expression levels are shown as log_2(FPKM + 1). (b) Principal component analysis (PCA) of all samples. Different colors represent different samples. (c) Distribution of reads mapped to genomic functional regions, including CDS, 3′UTR, intron, and intergenic regions. Different colors indicate different genomic features. (d) Heatmap of sample correlation. Color gradient from blue to red indicates low to high correlation.

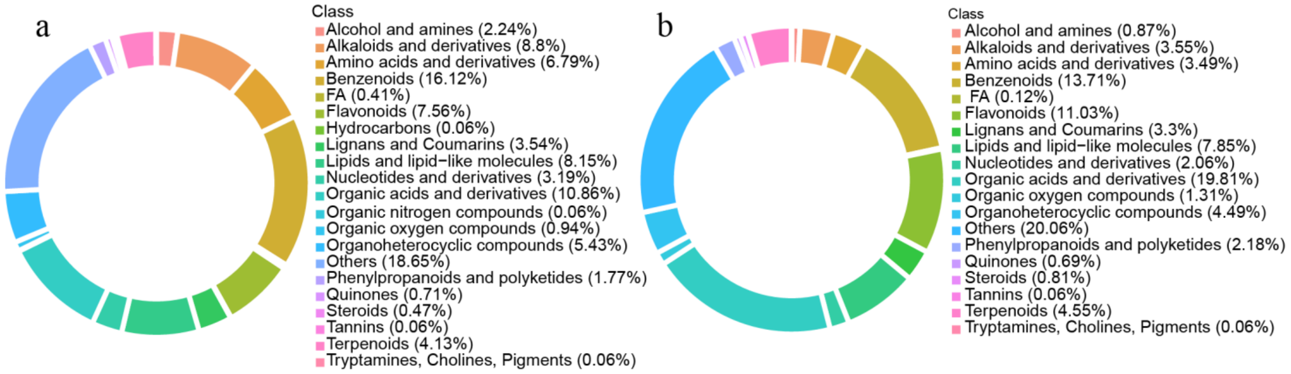

Figure S2. Metabolite class composition donut plots. (a) Positive ion mode; and (b) Negative ion mode. Each color represents a distinct metabolite class, and the area of each segment indicates its relative abundance.

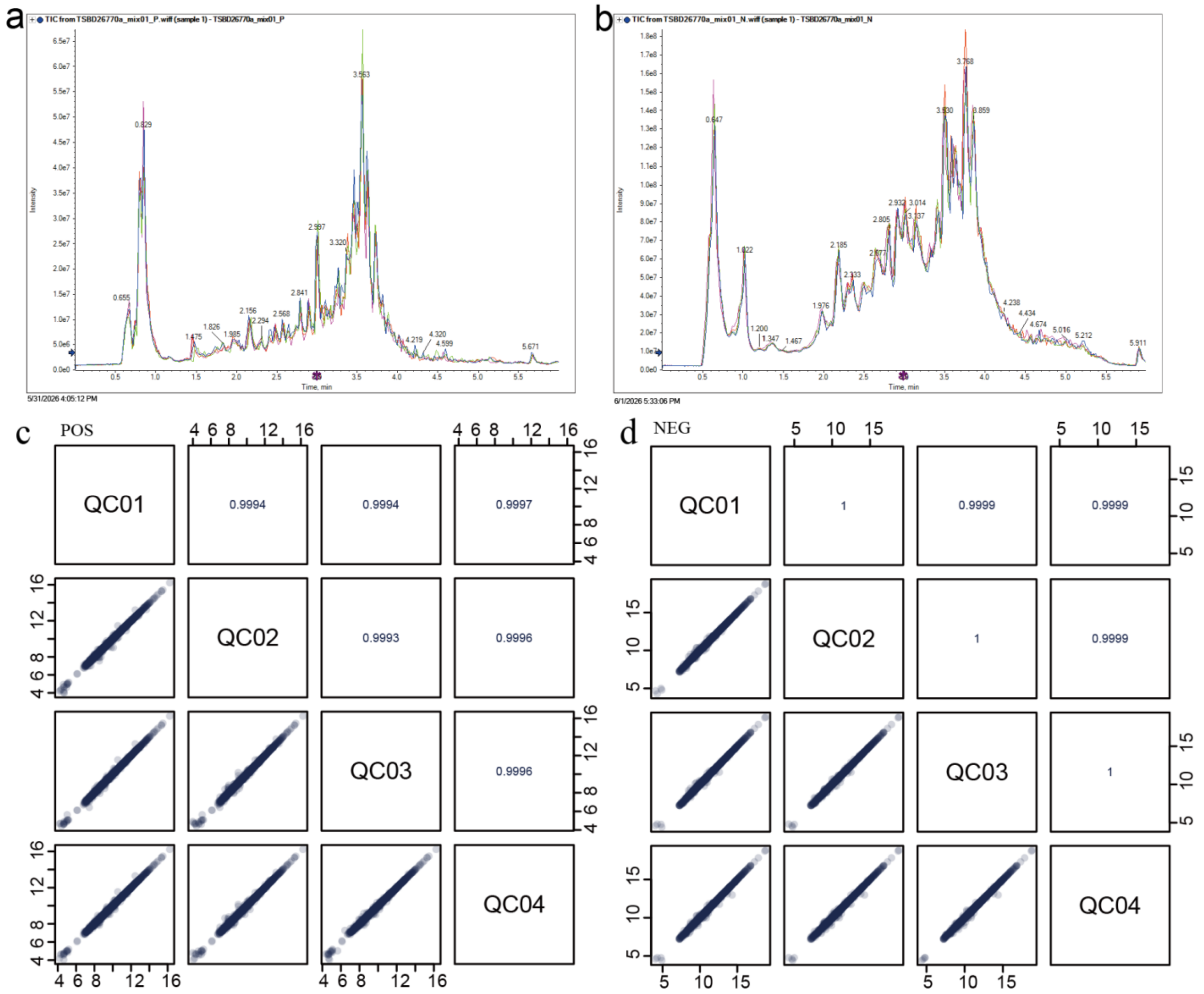

Figure S3. Quality control (QC) assessment of samples. (a) Total ion chromatograms (TIC) overlay of QC samples in positive ion mode; (b) TIC overlay of QC samples in negative ion mode; (c) correlation plot of QC samples in positive ion mode; (d) correlation plot of QC samples in negative ion mode.

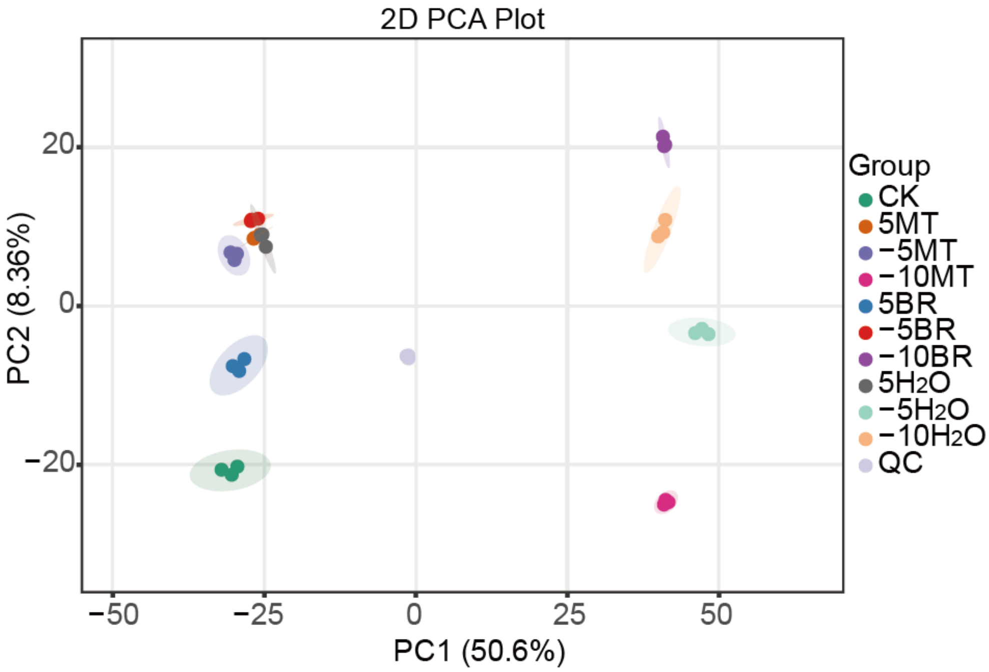

Figure S4. PCA score plot of mass spectrometry data from all sample groups and QC samples. PC1, PC2, and PC3 represent the first, second, and third principal components, respectively, and the percentages indicate the explained variance of each component in the dataset. Each point represents an individual sample. Samples from the same group are shown in the same color, and "Group" indicates the sample grouping.

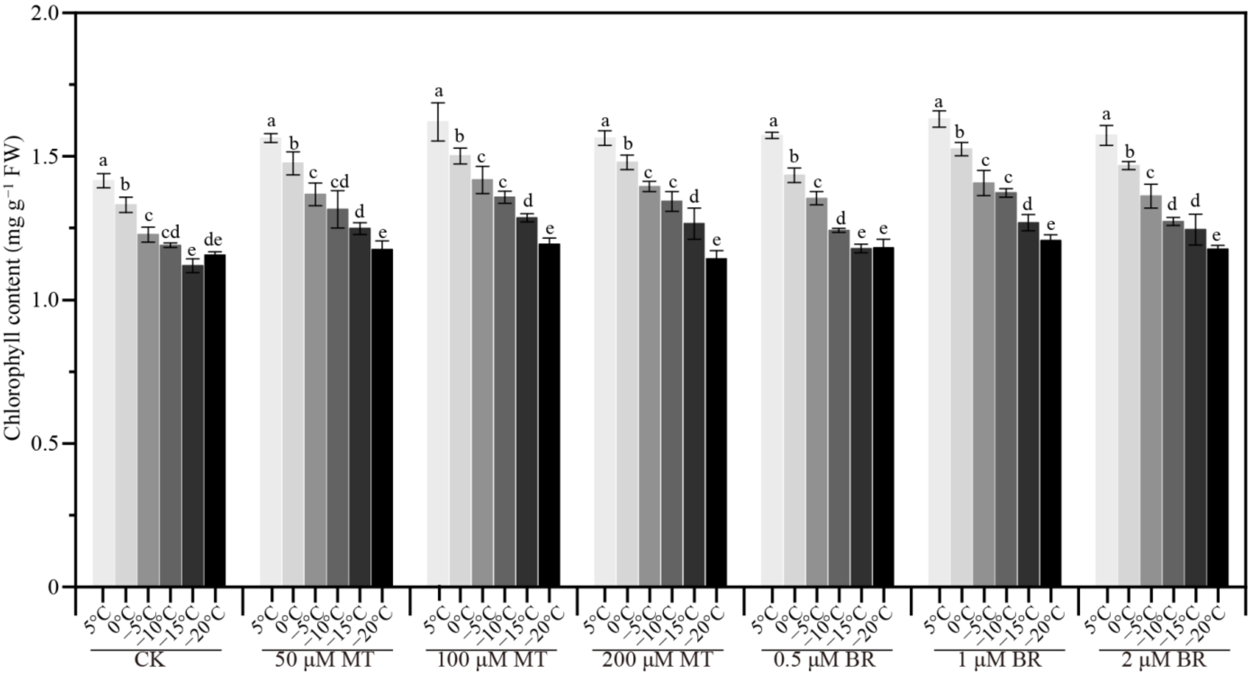

Figure S5. Chlorophyll content. Data are presented as mean ± standard deviation (SD). Different lowercase letters indicate statistically significant differences among treatments.
